# Supplementary material for: Mechanical Properties of Fused Deposition Modeling of Polyetheretherketone (PEEK) and Interest for Dental Restorations: A Systematic Review
Source: Materials (Basel). 2022 Sep 30;15(19):6801. doi: 10.3390/ma15196801 (PMC9572506; doi:10.3390/ma15196801)
Supplement: Supplementary file 1 [file materials-15-06801-s001.zip › materials-1876417-SI.pdf]

Table S1

1. Abdel Hay, J.; Smayra, T.; Moussa, R. Customized Polymethylmethacrylate Cranioplasty Implants Using 3-Dimensional Printed Polylactic Acid Molds: Technical Note with 2 Illustrative Cases. *World Neurosurg*. **2017**, *105*, 971-979.

2. Abdelaziz, M.S.; Tella, E. Digital fabrication of polyetheretherketone retentive bar attachment inserts as overdenture maintenance: A dental technique. *J. Prosthet. Dent*. **2022.**

3. Abdelaziz, M.; Fawzy, A.; Ghali, R.; Nassar, H. Retention of Different Attachment Systems for Digitally Designed Mandibular Implant Overdenture. *J. Prosthodont.* **2022.**

4. Abdullah, F.; Okuyama, K.; Morimitsu, A.; Yamagata, N. Effects of Thermal Cycle and Ultraviolet Radiation on 3D Printed Carbon Fiber/Polyether Ether Ketone Ablator. *Aerospace*. **2020**, 7.

5. Adl Amini, D.; Moser, M.; Oezel, L.; et al. Early Outcomes of Three-Dimensional–Printed Porous Titanium versus Polyetheretherketone Cage Implantation for Stand-Alone Lateral Lumbar Interbody Fusion in the Treatment of Symptomatic Adjacent Segment Degeneration. *World Neurosurg*. **2022**, 162, e14-e20.

6. Adl Amini, D.; Okano, I.; Oezel, L.; et al. Evaluation of cage subsidence in standalone lateral lumbar interbody fusion: Novel 3D-printed Titanium versus Polyetheretherketone (PEEK) Cage. *Eur Spine J*. **2021**, 30, 3403-3404.

7. Adrian-Martinez, S.; Ageron, M.; Aiello S.;et al. A method to stabilise the performance of negatively fed KM3NeT photomultipliers. *J Instrument*. **2016**, 11.

8. Ahmad, A.F.; Yaakob, H.; Khalil, A.; Georges, P. Evaluating patients' satisfaction level after using 3D printed PEEK facial implants in repairing maxillofacial deformities. *Ann Med Surg (Lond)*. **2022**, 79, 104095.

9. Ahn, H.; Patel,R.R.; Hoyt, A.J.; et al. Biological evaluation and finite-element modeling of porous poly(para-phenylene) for orthopaedic implants. *Acta Biomater*. **2018**, 72, 352-361.

10. Akl, M.; Stendahl, C. Removable Partial Denture Frameworks in the Age of Digital Dentistry: A Review of the Literature. *Prosthesis*. **2022**, 4, 184-201.

11. Al Mortadi, N.; Al Husein, B.; Alzoubi, K.; Khabour, O.; Eggbeer, D. Cytotoxicity of 3D Printed Materials for Potential Dental Applications: An In Vitro Study. *Open Dent. J.* **2022**, 16.

12. Al-Mortadi, N.; Bataineh, K.; Albakri, I. A Three – Dimensional Finite Element Analysis of Polyetheretherketone PEEK in Dental Implant Prosthesis: A Novel Implant System. *Open Dent J*. **2022,** 16.

13. AlAlaween, W.; Abueed, O.; Gharaibeh, B.; et al. The development of a radial based integrated network for the modelling of 3D fused deposition. *Rapid Prototyp. J.*

14. Alam, F.; Varadarajan, K.; Koo, J.; Wardle, B.; Kumar, S. Additively Manufactured Polyetheretherketone (PEEK) with Carbon Nanostructure Reinforcement for Biomedical Structural Applications. *Adv. Eng. Mater.* **2020**, 22.

15. Alan, N.; Vodovotz, L.; Muthiah, N.; et al. Subsidence after lateral lumbar interbody fusion using a 3D-printed porous titanium interbody cage: single-institution case series. *J. Neurosurg. Spine*. **2022**, 1-7.

16. Alauddin, M.S. A review of polymer crown materials: Biomechanical and material science. *J. Clin. Diagn. Res*. **2019,** 13, ZE01-ZE05.

17. Algarni, M.; Ghazali, S. Comparative Study of the Sensitivity of PLA, ABS, PEEK, and PETG's Mechanical Properties to FDM Printing Process Parameters. *Crystals.* **2021**, 11.

18. Alimi, O.; Meijboom, R. Current and future trends of additive manufacturing for chemistry applications: a review. *J. Mater. Sci*. **2021**, 56, 16824-16850.

19. Alkhaibary, A.; Alharbi, A.; Alnefaie, N.; Aloraidi, A.; Khairy, S. Cranioplasty: A Comprehensive Review of the History,

Materials, Surgical Aspects, and Complications. *World Neurosurg*. **2020**, 139, 445-452.

20. Alvarez, J.E.; Snijder, H.; Vaneker, T.; et al. Visco-elastic sintering kinetics in virgin and aged polymer powders. *Powder Technol*. **2022**, 397.

21. Amini, D.A.; Oezel, L.; Okano, I.; et al. Evaluation of cage subsidence in standalone lateral lumbar interbody fusion: Novel 3D printed titanium versus polyetheretherketone (PEEK) cage. *Spine J*. **2021**, 21, S37.

22. Andrew, J.; Alhashmi, H.; Schiffer, A.; Kumar, S.; Deshpande, V. Energy absorption and self-sensing performance of 3D printed CF/PEEK cellular composites. *Mater. Des.* **2021**, 208.

23. Ansari, M. Bone tissue regeneration: biology, strategies and interface studies. *Prog. Biomater*. **2019**, 8, 223-237.

24. Arif, M.; Alhashmi, H.; Varadarajan, K.; Koo, J.; Hart, A.; Kumar, S. Multifunctional performance of carbon nanotubes and graphene nanoplatelets reinforced PEEK composites enabled via FFF additive manufacturing. *Compos.B Eng.* **2020**, 184.

25. Arif, M.; Kumar, S.; Varadarajan, K.; Cantwell, W. Performance of biocompatible PEEK processed by fused deposition additive manufacturing. *Mater. Des*. **2018,** 146, 249-259.

26. Arts, M.; Torensma, B.; Wolfs, J. Porous titanium cervical interbody fusion device in the treatment of degenerative cervical radiculopathy; 1-year results of a prospective controlled trial. *Spine J*. **2020,** 20, 1065-1072.

27. Ashraf, M.; Choudhary, N.; Kamboh, U.A.; et al. Early experience with patient-specific low-cost 3D-printed polymethylmethacrylate cranioplasty implants in a lower-middle-income-country: Technical note and economic analysis. *Surg. Neurol. Int*. **2022**, 13, 270.

28. Atamian, E.K.; Hazkour, N.; Palacios, J.; et al. The Use of the Three-Dimensional Printed Polyether Ether Ketone Implant in Secondary Craniosynostosis Revision. *J. Craniofac. Surg*. **2022**, 33, 1734-1738.

29. Baek, I.; Kwon, O.; Lim, C.M.; Park, K.Y., Bae, C.J. 3D PEEK Objects Fabricated by Fused Filament Fabrication (FFF). *Materials (Basel)*. **2022**, 15.

30. Bagaria, V.; Bhansali, R.; Pawar, P. 3D printing- creating a blueprint for the future of orthopedics: Current concept review and the road ahead! *J. Clin. Orthop. Trauma*. **2018**, 9, 207-212.

31. Bai, C.; Ji, M.; Qin, D.; Wan, M. A 3D-printed skull model with corresponding acoustic characteristic of human skull for ultrasound brain imaging and diagnosis. *J. Ther. Ultrasound*. **2018**, 6.

32. Baldia, M.; Joseph, M.; Sharma, S.; et al. Customized cost-effective polymethylmethacrylate cranioplasty: a cosmetic comparison with other low-cost methods of cranioplasty. *Acta Neurochir.* **2022**, 164, 655-667.

33. Baligidad, S.; Kumar, G.; Maharudresh, A.; Lekshmi, I.; Rajasree, S.; Pillai, R. Investigation on strain rate sensitivity of 3D printed sPEEK-HAP/ rGO composites. *J. Manufact. Proc.* **2022**, 79, 789-802.

34. Banerjee, K.; Debroy, M.; Balla, V.; Bodhak, S. Recent progress in 3D-printed polyaryletherketone (PAEK)-based high-performance polymeric implants for musculoskeletal reconstructions. *J. Mater. Res*. **2021**, 36, 3877-3893.

35. Baptista, L.S.; Kronemberger, G.S.; Côrtes, I. ; et al. Adult stem cells spheroids to optimize cell colonization in scaffolds for cartilage and bone tissue engineering. *Int. J. Mol. Sci*. **2018**, 19.

36. Barragan-Paredes, M.; Mosquera-Victoria, I.; Viveros-Rebolledo, C.; Rodriguez-Paz, M.; Munoz-Velez, M.; Valencia-Llano, C. Comparison of the Mechanical Properties of Temporary Abutments Made of Polyetheretherketone and Photopolymeric Resin. *Open Dent. J*. **2021**, 15, 512-519.

37. Barros, A.D.; Brauge, D.; QuÉhan, R.; Cavallier, Z.; Roux, F.E.;, Moyse, E. One-Step Customized PEEK Cranioplasty After 3D Printed Resection Template Assisted Surgery for a Frontal Intraosseous Meningioma: A Case Report. *Turk. Neurosurg*. **2021,** 31, 142-147.

38. Bartolomeu, F.; Buciumeanu, M.; Costa, M.M.; et al. Multi-material Ti6Al4V & PEEK cellular structures produced by Selective Laser Melting and Hot Pressing: A tribocorrosion study targeting orthopedic applications. *J. Mech. Behav. Biomed. Mater*. **2019**, 89, 54-64.

39. Basgul, C.; MacDonald, D.W.; Siskey, R.; Kurtz, S.M. Thermal Localization Improves the Interlayer Adhesion and Structural Integrity of 3D printed PEEK Lumbar Spinal Cages. *Materialia (Oxf)*. **2020**, 10, 100650.

40. Basgul, C.; Spece, H.; Sharma, N.; Thieringer, F.M.; Kurtz, S.M. Structure, properties, and bioactivity of 3D printed PAEKs for implant applications: A systematic review. *J. Biomed. Mater. Res. B Appl. Biomater*. **2021**, 109, 1924-1941.

41. Basgul, C.; Thieringer, F.M.; Kurtz, S.M. Heat Transfer-Based Non-isothermal Healing Model for the Interfacial Bonding Strength of Fused Filament Fabricated Polyetheretherketone. *Addit. Manuf*. **2022**, 46, 102097.

42. Basgul, C.; Yu, T.; MacDonald, D.W.; Siskey, R.; Marcolongo, M.; Kurtz, S.M. Structure-Property Relationships for 3D printed PEEK Intervertebral Lumbar Cages Produced using Fused Filament Fabrication. *J. Mater. Res*. **2018**, 33, 2040-2051.

43. Basgul, C.; Yu, T.; MacDonald, D.W.; Siskey, R.; Marcolongo, M.; Kurtz, S.M. Does annealing improve the interlayer adhesion and structural integrity of FFF 3D printed PEEK lumbar spinal cages? *J. Mech. Behav. Biomed. Mater*. **2020**, 102, 103455.

44. Beard, R.; Van Horn, M.R.; Bucklen, B. 3D-printed porous biomimetic titanium promotes osteogenic gene expression in murine mesenchymal stem cells. *Spine J*. **2020,** 20, S32-S33.

45. Berretta, S.; Davies, R.; Shyng, Y.; Wang, Y.; Ghita, O. Fused Deposition Modelling of high temperature polymers: Exploring CNT PEEK composites. *Polym. Test.* **2017**, 63, 251-262.

46. Berretta, S.; Evans, K.; Ghita, O. Additive manufacture of PEEK cranial implants: Manufacturing considerations versus accuracy and mechanical performance. *Mater. DES*. **2018**, 139, 141-152.

47. Bhandari, S.; Lopez-Anido, R. Discrete-Event Simulation Thermal Model for Extrusion-Based Additive Manufacturing of PLA and ABS. *Materials*. **2020**, 13.

48. Bhargava, K.C.; Thompson, B.; Malmstadt, N. Discrete elements for 3D microfluidics. *Proc. Natl. Acad. Sci. U S A*. **2014**, 111, 15013-15018.

49. Birkelid, A.H.; Eikevåg, S.W.; Elverum, C.W.; Steinert, M. High-performance polymer 3D printing - Open-source liquid cooled scalable printer design. *HardwareX*. **2022**, 11, e00265.

50. Bishop, G.W.; Satterwhite, J.E.; Bhakta, S.; et al. 3D-Printed Fluidic Devices for Nanoparticle Preparation and Flow-Injection Amperometry Using Integrated Prussian Blue Nanoparticle-Modified Electrodes. *Anal. Chem*. **2015**, 87, 437-5443.

51. Bloise, N.; Waldorff, E.I.; Montagna, G.; et al. Early Osteogenic Marker Expression in hMSCs Cultured onto Acid Etching-Derived Micro- and Nanotopography 3D-Printed Titanium Surfaces. *Int. J. Mol. Sci*. **2022**, 23.

52. Bochkareva, S.; Alexenko, V.; Lyukshin, B.; Buslovich, D.; Panin, S. Effect of the Thermal Conductivity of Mated Materials on the Wear Intensity of a Polymerpolymer Friction Pair. *Mechanics of composite materials*. **2022**, 58, 307-318.

53. Bogu, V.P.; Kumar, Y.R.; Kumar Khanara, A. Modelling and structural analysis of skull/cranial implant: beyond mid-line deformities. *Acta Bioeng. Biomech*. **2017**, 19, 125-131.

54. Brill, S.H.; Chong, J.H.; Kim, D.; Cho, W. Spine Surgery—Part I: Biomechanics, Materials, and 3-D Printing Technology: Surgical Perspective and Clinical Impact. *Springer Ser Biomater. Sci. Eng*. **2022**, 18, 209-229.

55. Brinkhues, S.; Kanthamneni, A.; Brose, A.; Majcherek, S.; Schmidt, B. Investigation of Adhesion Strength of Metallization on Thermoplastic and Ceramic Substrates. *2016 12th International Congress Molded Interconnect Devices (MID)*, **2016**, 94-99.

56. Buican, G.; Zaharia, S.; Pop, M. et al. Fabrication and Characterization of Fiber-Reinforced Composite Sandwich Structures Obtained by Fused Filament Fabrication Process. *Coatings*. **2021**, 11.

57. Caldona, E.; Dizon, J.; Viers, R.; Garcia, V.; Smith, Z.; Advincula, R. Additively manufactured high-performance polymeric materials and their potential use in the oil and gas industry. *Mrs Communications*. **2021**, 11, 701-715.

58. Callanan, T.C.; Walker, B.; Grinberg, S.; Cammisa, F.P.; Abjornson, C. Prospective, 88 patient series study of the use of a novel 3-D printed titanium truss system cage in anterior cervical spinal surgery. *Spine J*. **2016**, 16, S360.

59. Carpenter, R.D.; Klosterhoff, B.S.; Torstrick, F.B.; et al. Effect of porous orthopaedic implant material and structure on load sharing with simulated bone ingrowth: A finite element analysis comparing titanium and PEEK. *J. Mech. Behav. Biomed. Mater*. **2018**, 80, 68-76.

60. Cattenone, A.; Morganti, S.; Alaimo, G.; Auricchio, F. Finite Element Analysis of Additive Manufacturing Based on Fused Deposition Modeling: Distortions Prediction and Comparison With Experimental Data. *J.Manuf. Sci.Eng.* **2019**, 141.

61. Challa, B.; Gummadi, S.; Elhattab, K.; Ahlstrom, J.; Sikder, P. In-house processing of 3D printable polyetheretherketone (PEEK) filaments and the effect of fused deposition modeling parameters on 3D-printed PEEK structures. *Int. J. Adv. Manuf. Technol.* **2022**, 121, 1675-1688.

62. Chan, J.L.; Bae, H.W.; Farber, S.H.; Uribe, J.S.; Eastlack, R.K.; Walker, C.T. Evolution of Bioactive Implants in Lateral Interbody Fusion. *Int. J. Spine Surg*. **2022**, 16, S61-S68.

63. Chandegra, R.; Pabla, R.; Perry, M.; Technological innovations in maxillofacial surgical trauma management. *Trauma*. **2015**, 17, 308-309.

64. Chang, B.; Li, X.; Parandoush, P.; Ruan, S.; Shen, C.; Lin, D. Additive manufacturing of continuous carbon fiber reinforced poly-ether-ether-ketone with ultrahigh mechanical properties. *Polym. Test.* **2020**, 88.

65. Chang, T.; Hsu, C.; Zhou, S.; Tsai, P.; Wang, F. Design of 3D Printed Porous Additive Manufactured Cages Using a Computer Model of T10-S1 Multilevel Spine. *2017 IEEE/SICE International Symposium on System Integration (SII).* **2017**, 475-480.

66. Chao, W.; Kolski-Andreaco, A. 3D Virtual Navigation in the Human Brain, Simulation of Planet Formation, and Bioprinting Cultured Cartilage Grafts. *Jove-Journal of Visualized Experiments.* **2014**.

67. Chavez, L.; Ibave, P.; Hassan, M. et al. Low-temperature selective laser sintering 3D printing of PEEK-Nylon blends: Impact of thermal post-processing on mechanical properties and thermal stability. *J.Appl. Polym.Sci*. **2022**, 139.

68. Chen, C.; Hao, Y.; Bai, X. et al. 3D printed porous Ti6Al4V cage: Effects of additive angle on surface properties and biocompatibility; bone ingrowth in Beagle tibia model. *Mater. Des*. **2019**, 175.

69. Chen, C.; Yin, Y.; Xu, H.; Li, Z.; Wang, F.; Chen, G. Personalized three-dimensional printed polyether-ether-ketone prosthesis for reconstruction after subtotal removal of chronic clavicle osteomyelitis: A case report. *Medicine (Baltimore)*. **2021**, 100, e25703.

70. Chen, J.; Cao, G.; Li, L.; Cai, Q.; Dunne, N.; Li, X. Modification of polyether ether ketone for the repairing of bone defects. *Biomed. Mater*. **2022**, 17.

71. Chen, P.; Su, J.; Wang, H. et al. Aging mechanism of polyetheretherketone powder during layer-wise infrared radiation of high-temperature laser powder bed fusion. *Mater. Des.* **2022**, 213.

72. Chen, P.; Su, J.; Wang, H. et al. Mechanical properties and microstructure characteristics of lattice-surfaced PEEK cage fabricated by high-temperature laser powder bed fusion. *J. Mater. Sci. Technol.* **2022**, 125, 105-117.

73. Chen, P.; Wang, H.; Su, J. et al. Recent Advances on High-Performance Polyaryletherketone Materials for Additive Manufacturing. *Adv. Mater.* **2022**.

74. Chen, P.; Wu, H.; Yang, L. et al. A 2D correlation infrared spectroscopic study on the temperature-induced molecular motion mechanism concerning self-formed composite structure of 3D printed PA6. *Polymer.* **2019**, 167, 48-53.

75. Chen, S.; Yang, J.; Li, K.; Lu, B.; Ren, L. Carboxylic acid-functionalized TiO2 nanoparticle-loaded PMMA/PEEK copolymer

matrix as a dental resin for 3D complete denture manufacturing by stereolitographic technique. *Int. J. Food Prop.* **2018**, 21, 2557-2565.

76. Chen, S.G.; Yang, J.; Jia, Y.G.; Lu, B.; Ren, L. TiO(2) and PEEK Reinforced 3D Printing PMMA Composite Resin for Dental Denture Base Applications. *Nanomaterials (Basel)*. **2019**, 9.

77. Chen, W.; Zhang, X.; Tan, D. et al. Improvement in Mechanical Properties of 3D-Printed PEEK Structure by Nonsolvent Vapor Annealing. *Macromol. Rapid Commun*. **2022**, 43, e2100874

78. Chen, X.; Wu, Y.; Liu, H. et al. Mechanical performance of PEEK-Ti6Al4V interpenetrating phase composites fabricated by powder bed fusion and vacuum infiltration targeting large and load-bearing implants. *Mater. Des.* **2022**, 215.

79. Chen, X.; Wang, F.; Sun, F.; Zhang, L.; Wu, G. Digital fabrication of an adult speech aid prosthesis by using a 3-dimensionally printed polyetheretherketone framework. *J. Prosthet. Dent*. **2022**, 127, 358-361

80. Chen, Z.; Tong, J.; Li, X.; Yan, Y. Advantages and disadvantages of interbody implant materials in lumbar fusion. *Chin. J. Tissue Eng. Res*. **2022**, 26, 1671-1678.

81. Cheng, B.C.; Jaffee, S.; Averick, S.; Swink, I.; Horvath, S.; Zhukauskas, R. A comparative study of three biomaterials in an ovine bone defect model. *Spine J*. **2020**, 20, 457-464.

82. Cheng, K.; Liu, Y.; Wang, R. et al. Topological optimization of 3D printed bone analog with PEKK for surgical mandibular reconstruction. *J. Mech. Behav. Biomed. Mater.* **2020**, 107.

83. Cheng, L.; Shoma Suresh, K.; He, H. et al. 3d printing of micro-and nanoscale bone substitutes: A review on technical and translational perspectives. *Int. J. Nanomed*. **2021**, 16, 4289-4319

84. Cho, M.; Kane, A.; Hallac, R.; Gangopadhyay, N.; Seaward, J. Liquid Latex Molding: A Novel Application of 3D Printing to Facilitate Flap Design. *Cleft Palate Craniofac. J.* **2017**, 54, 453-456.

85. Choudhury, S.; Pandey, M.; Bhattacharya, S. Recent Developments in Surface Modification of PEEK Polymer for Industrial Applications: A Critical Review. *Rev Adhes Adhes.* **2021**, 9, 401-433.

86. Chumaevskii, A.; Ivanov, A.; Filippov, A.; Rubtsov, V.; Kolubaev, E. Tribological Dry Sliding Behavior of Chopped Carbon Fiber Reinforced Polyetheretherketone. *AIP Conference Proceedings 1909, 020025*. **2017**.

87. Cicala, G.; Recca, G.; Carbone, D.; Pergolizzi, E.; Lorusso, A. Novel engineering polymers for additive manufacturing. *J. Appl. Biomater. Funct. Mater*. **2016**, 14, e344.

88. Cicala, G.; Latteri, A.; Del Curto, B.; Lo Russo, A.; Recca, G.; Farè, S. Engineering Thermoplastics for Additive Manufacturing: A Critical Perspective with Experimental Evidence to Support Functional Applications. *J. Appl. Biomater. Funct. Mater*. **2017**, 15, 10-18.

89. Collier, J.; Gore, S.; Dawood, A.; Kirkpatrick, N.; Eccles, S. Custom-made facial onlay implants: Our experience of 50 consecutive cases. *Br. J. Oral Maxillofac. Surg*. **2017**, 55, e85.

90. Collinson, D.; von Windheim, N.; Gall, K.; Brinson, L. Direct evidence of interfacial crystallization preventing weld formation during fused filament fabrication of poly(ether ether ketone). *Addit. Manuf.* **2022**, 51.

91. Comelli, C.; Davies, R.; van der Pol, H.; Ghita, O. PEEK filament characteristics before and after extrusion within fused filament fabrication process. *J. Mater. Sci.* **2022**, 57, 766-788.

92. Copperman, T.S.; Idowu, O.O.; Jalaj, S. et al. Patient-Specific Implants in Oculofacial Plastic Surgery. *Ophthalmic Plast. Reconstr. Surg*. **2021**, 37, 241-247.

93. Corea, J.; Ye, P.P.; Arias, A.C.; Pauly, K.B.; Lustig, M. Acoustically transparent surface coil arrays for MR guided HIFU. *J. Ther. Ultrasound*. **2016**, 4.

94. Corso, K.A.; Kothari, P.; Corrado, K.; Michielli, A.; Ruppenkamp, J.; Bowden, D. Early revision events among patients with a three dimensional (3D) printed cellular titanium or PEEK (polyetheretherketone) spinal cage for single-level lumbar spinal fusion. *Expert Rev. Med. Devices*. **2022**, 19, 195-201.

95. Costa, M.M.; Lima, R.; Alves, N. et al. Multi-material cellular structured orthopedic implants design: In vitro and bio-tribological performance. *J. Mech. Behav. Biomed. Mater*. **2022**, 131.

96. Cotton, J.; Menzel, F.; Ziegler, T.; Pichler, B.; Neumaier, J.; Maurer, A. Evaluation of an open-source 3D printed microfluidic system for the production of radiopharmaceuticals. *Nucl. Med. Biol*. **2022**, 108-109, S123-S124.

97. Cuzzocrea, F.; Ghiara, M.; Gaeta, M.; Fiore, M.R.; Benazzo, F.; Gentile, L. Carbon fiber screws in spinal tumor and metastasis: Advantages in surgery, radio-diagnostic and hadrontherapy. *J. Biol. Regul. Homeostatic Agents*. **2019**, 33, 1265-1268.

98. Czyżewski, W.; Jachimczyk, J.; Hoffman, Z. et al. Low-Cost Cranioplasty-A Systematic Review of 3D Printing in Medicine. *Materials (Basel)*. **2022**, 15.

99. Dai, Y.; Guo, H.; Chu, L. et al. Promoting osteoblasts responses in vitro and improving osteointegration in vivo through bioactive coating of nanosilicon nitride on polyetheretherketone. *J. Orthop. Transl*. **2020**, 24, 198-208.

100. Das, A.; Chatham, C.; Fallon, J. et al. Current understanding and challenges in high temperature additive manufacturing of engineering thermoplastic polymers. *Addit. Manuf*. **2020**, 34.

101. Day, K.M.; Phillips, P.M.; Sargent, L.A. Correction of a Posttraumatic Orbital Deformity Using Three-Dimensional Modeling, Virtual Surgical Planning with Computer-Assisted Design, and Three-Dimensional Printing of Custom Implants. *Craniomaxillofacial Trauma Reconstr*. **2018**, 11, 78-82.

102. de Avila, E.; Eo, J.; Kim, J.; Kim, N. Heat Treatment Effect on Mechanical Properties of 3D Printed Polymers. MATEC Web Conf. **2019**, 264.

103. de Leon, A.; da Silva, I.; Pangilinan, K.; Chen, Q.; Caldona, E.; Advincula, R. High performance polymers for oil and gas applications. *React. Funct Polym.* **2021**, 162.

104. Delaney, L.; Sevit, A.; Eisenbrey, J. et al. Characterization of Ultrasound-Triggered Bulk Antibiotic Release from Novel Spinal Hardware. *2019 IEEE International Ultrasonics Symposium (IUS).* **2019**, 231-234.

105. Deng, L.; Deng, Y.; Xie, K. AgNPs-decorated 3D printed PEEK implant for infection control and bone repair. *Colloids Surf. B Biointerfaces*. **2017**, 160, 483-492.

106. Deng, X.; Zeng, Z.; Peng, B.; Yan, S.; Ke, W. Mechanical Properties Optimization of Poly-Ether-Ether-Ketone via Fused Deposition Modeling. *Materials (Basel)*. **2018**, 11.

107. Deng, Y.; Shi, X.; Chen, Y. et al. Bacteria-Triggered pH-Responsive Osteopotentiating Coating on 3D-Printed Polyetheretherketone Scaffolds for Infective Bone Defect Repair. *Ind. Eng. Chem. Res*. **2020**, 59, 12123-12135.

108. Denholm, K.A.; Pilkington, R.J.J.; Keith, D.J.W. Silent sinus syndrome: Systematic literature review and report of case treated with a customised peek orbital floor implant. *Br. J. Surg*. **2021**, 108, vi167-vi168.

109. Ding, L.; Chen, X.; Zhang, J.; Wang, R.; Wu, G. Digital fabrication of a maxillary obturator prosthesis by using a 3-dimensionally-printed polyetheretherketone framework. *J. Prosthet. Dent*. **2021**.

110. Ding, L.; Lu, W.; Zhang, J.; Yang, C.; Wu, G. Preparation and Performance Evaluation of Duotone 3D-Printed Polyetheretherketone as Oral Prosthetic Materials: A Proof-of-Concept Study. *Polymers (Basel)*. **2021**, 13.

111. Ding, S.; Zou, B.; Wang, P.; Ding, H. Effects of nozzle temperature and building orientation on mechanical properties and microstructure of PEEK and PEI printed by 3D-FDM. *Polym. Test.* **2019**, 78.

112. DiNoro, J.N.; Paxton, N.C.; Skewes, J. et al. Laser Sintering Approaches for Bone Tissue Engineering. *Polymers (Basel)*. **2022**, 14.

113. Diouf-Lewis, A.; Farahani, R.; Iervolino, F. et al. Design and characterization of carbon fiber-reinforced PEEK/PEI blends for Fused Filament Fabrication additive manufacturing. *Mater. Today Commun.* **2022**, 31.

114. Dizon, J.; Gache, C.; Cascolan, H.; Cancino, L.; Advincula, R. Post-Processing of 3D-Printed Polymers. *Technologies*. **2021**, 9.

115. Doh, G.; Eo, S.; Hong, K.Y. Temporal Hollowing Augmentation With Polyetheretherketone Patient-Specific Implant. *J. Craniofac. Surg*. **2019**, 30, 2131-2133.

116. Domingues, T.; Cachaco, A.; Sousa, P. et al. Development of a custom setup for additive manufacturing of high-performance thermoplastics. **2022**, 847-856.

117. Dose, T.; Krüger, D.; Hammel, J.U. et al. A modular in situ device for stress testing of biomaterials. *Biomed. Tech.* **2017**, 62, 114-115.

118. Dou, H.; Ye, W.; Zhang, D.; Cheng, Y.; Tian, Y. Compression Performance with Different Build Orientation of Fused Filament Fabrication Polylactic Acid, Acrylonitrile Butadiene Styrene, and Polyether Ether Ketone. *J. Mater. Eng. Perform.* **2022**, 31, 1925-1933.

119. Du, Z.; Yu, X.; Nie, B. et al. Effects of magnesium coating on bone-implant interfaces with and without polyether-ether-ketone particle interference: A rabbit model based on porous Ti6Al4V implants. *J. Biomed. Mater. Res. B Appl. Biomater*. **2019**, 107, 2388-2396.

120. Dua, R.; Rashad, Z.; Spears, J.; Dunn, G.; Maxwell, M. Applications of 3D-Printed PEEK via Fused Filament Fabrication: A Systematic Review. *Polymers (Basel)*. **2021**, 13.

121. Duan, X.; Wang, B.; Yang, L.; Kadakia, A.R. Applications of 3D Printing Technology in Orthopedic Treatment. *BioMed. Res. Int*. **2021**.

122. Dunn, K.L.; Dunn, K.H.; Hammond, D.; Lo, S. Three-dimensional printer emissions and employee exposures to ultrafine particles during the printing of thermoplastic filaments containing carbon nanotubes or carbon nanofibers. *J. Nanopart. Res*. **2020**, 22.

123. Dux, K.E. Implantable Materials Update. *Clin Podiatr Med Surg*. **2019**, 36, 535-542.

124. Eblenkamp, M.; Fischer, S.; Pammer, S.; Leonhardt, S. Additive manufactured multimicrophasic (MMP) systems for biomedical applications-perspectives and concepts. *Biomed. Tech*. **2017**, 62.

125. El Magri, A.; El Mabrouk, K.; Vaudreuil, S.; Chibane, H.; Touhami, M. Optimization of printing parameters for improvement of mechanical and thermal performances of 3D printed poly(ether ether ketone) parts. *J Appl. Polym. Sci*. **2020**, 137.

126. El Magri, A.; El Mabrouk, K.; Vaudreuil, S.; Touhami, M. Experimental investigation and optimization of printing parameters of3Dprinted polyphenylene sulfide through response surface methodology. *J Appl Polym Sci*. **2021**, 138.

127. El Magri, A.; El Mabrouk, K.; Vaudreuil, S. Preparation and characterization of poly(ether ether ketone)/poly(ether imide) [PEEK/PEI] blends for fused filament fabrication. *J Mater. Sci.***2021**, 56, 14348-14367.

128. El Magri, A.; Vanaei, S.; Vaudreuil, S. An overview on the influence of process parameters through the characteristic of 3D-printed PEEK and PEI parts. *High Perform. Polym.* **2021**, 33, 862-880.

129. Eley, K.A.; Moazen, M.; Delso, G. et al. Enhancing Distraction Osteogenesis With Carbon Fiber Reinforced Polyether Ether Ketone Bone Pins and a Three-Dimensional Printed Transfer Device to Permit Artifact-Free Three-Dimensional Magnetic Resonance Imaging. *J. Craniofac. Surg*. **2021**, 32, 360-364.

130. Elhattab, K.; Sikder, P.; Walker, J.; Bottino, M.; Bhaduri, S. Fabrication and evaluation of 3-D printed PEEK scaffolds containing Macropores by design. *Mater. Lett.* **2020**, 263.

131. Eltorai, A.E.M.; Nguyen, E.; Daniels, A.H. Three-dimensional printing in orthopedic surgery. *Orthopedics*. **2015**, 38, 684-687.

132. Erkal, J.L.; Selimovic, A.; Gross, B.C. et al. 3D printed microfluidic devices with integrated versatile and reusable electrodes. *Lab. Chip*. **2014**, 14, 2023-2032.

133. Eshkalak, S.; Ghomi, E.; Dai, Y.; Choudhury, D.; Ramakrishna, S. The role of three-dimensional printing in healthcare and medicine. *Mater. Des.* **2020**, 194.

134. Fairag, R.; Li, L.; Ramirez-GarciaLuna, J.L. et al. A Composite Lactide-Mineral 3D-Printed Scaffold for Bone Repair and Regeneration. *Front. Cell Dev. Biol*. **2021**, 9, 654518.

135. Fan, D.; Li, Y.; Wang, X. et al. Progressive 3D Printing Technology and Its Application in Medical Materials. *Front. Pharmacol*. **2020**, 11.

136. Fang, G.; Sang, H. In Reply to the Letter to the Editor Regarding "Biomechanical Comparison of Stand-Alone and Bilateral Pedicle Screw Fixation for Oblique Lumbar Interbody Fusion Surgery—A Finite Element Analysis." *World Neurosurg*. **2021**, 150, 250.

137. Feltz, K.P.; Macfadden, L.N.; Gieg, S.D.; Lough, C.P.; Bezold, W.A.; Skelley, N.W.M. Mechanical properties of 3D-printed orthopedic one-third tubular plates and cortical screws. *J. 3D Printing Med*. **2022**, 6, 129-145.

138. Feng, P.; Jia, J.; Peng, S.; Yang, W.; Bin, S.; Shuai, C. Graphene oxide-driven interfacial coupling in laser 3D printed PEEK/PVA scaffolds for bone regeneration. *Virtual and Phys. Prototyp.* **2020**, 15, 211-226.

139. Feng, X.; Ma, L.; Liang, H. et al. Osteointegration of 3D-Printed Fully Porous Polyetheretherketone Scaffolds with Different Pore Sizes. *ACS Omega*. **2020**, 5, 26655-26666.

140. Feng, X.; Yu, H.; Liu, H. et al. Three-Dimensionally-Printed Polyether-Ether-Ketone Implant with a Cross-Linked Structure and Acid-Etched Microporous Surface Promotes Integration with Soft Tissue. *Int. J. Mol. Sci*. **2019**, 20.

141. Ferrara, M.; Rinaldi, M.; Pigliaru, L.; Cecchini, F.; Nanni, F. Investigating the use of 3D printed soft magnetic PEEK-based composite for space compliant electrical motors. *J. Appl Polym. Sci.* **2022**, 139.

142. Fina, F.; Gaisford, S.; Basit, A.W. Powder bed fusion: The working process, current applications and opportunities. *AAPS Adv. Pharm. Sci. Ser*. **2018**, 31, 81-105.

143. Fogel, G.; Martin, N.; Williams, G.M. et al. Choice of Spinal Interbody Fusion Cage Material and Design Influences Subsidence and Osseointegration Performance. *World Neurosurg*. **2022**, 162, e626-e634.

144. Fournet, M.; Azaman, F.; Gunbay, S.; Chen, Y.; Devine, D. Orthopaedic 3D Printing in Orthopaedic Medicine. *In: Devine, D. (eds) Polymer-Based Additive Manufacturing. Springer, Cham.* **2019**, 142.

145. Francis, J.; Banerjee, I.; Chugh, A.; Singh, J.; Additive manufacturing of polyetheretherketone and its composites: A review. *Polym. Comp.* **2022**.

146. Freudenberg, W.; Wich, F.; Langhof, N.; Schaffoner, S. Additive manufacturing of carbon fiber reinforced ceramic matrix composites based on fused filament fabrication. *J. Eur. Ceram. Soc.* **2022**, 42, 822-1828.

147. Gao, R.; Xie, J.; Yang, J.; Zhuo, C.; Fu, J.; Zhao, P. Research on the Fused Deposition Modeling of Polyether Ether Ketone. *Polymers (Basel)*. **2021**, 13.

148. Gao, S.; Liu, R.; Xin, H.; Liang, H.; Wang, Y.; Jia, J. The Surface Characteristics, Microstructure and Mechanical Properties of PEEK Printed by Fused Deposition Modeling with Different Raster Angles. *Polymers (Basel)*. **2021**, 14.

149. Gao, X.; Wang, H.; Luan, S.; Zhou, G. Low-Temperature Printed Hierarchically Porous Induced-Biomineralization Polyaryletherketone Scaffold for Bone Tissue Engineering. *Adv. Health Mater*. **2022**, e2200977.

150. Gao, X.; Wang, H.; Zhang, X. et al. Preparation of Amorphous Poly(aryl ether nitrile ketone) and Its Composites with Nano Hydroxyapatite for 3D Artificial Bone Printing. *ACS Appl. Bio. Mater*. **2020**, 3, 7930-7940.

151. Garcia-Gonzalez, D.; Garzon-Hernandez, S.; Arias, A. A new constitutive model for polymeric matrices: Application to biomedical materials. *Compos. B Eng.* **2018**, 131, 117-129.

152. Garcia-Gonzalez, D.; Garzon-Hernandez, S.; Rusinek, A.; Bernier, R.; Arias, A. Low temperature mechanical behaviour of PVDF: cryogenic pre-treatment, quasi-static, cyclic and dynamic experimental testing and modelling. *Mech. Mater*. **2020**, 147.

153. Garcia-Gonzalez, D.; Rusinek, A.; Jankowiak, T.; Arias, A. Mechanical impact behavior of polyether-ether-ketone (PEEK). *Compos. Struct.* **2015**, 124, 88-99.

154. Garcia-Leiner, M.; Streifel, B.; Basgul, C.; MacDonald, D.; Kurtz, S. Characterization of polyaryletherketone (PAEK) filaments and printed parts produced by extrusion-based additive manufacturing. *Polym. Int.*. **2021**, 70, 1128-1136.

155. Genders, S.; Pichardo, S.; van Furth, W.; Notting, I. Custom-made 3D-printed polyetheretherketone (PEEK) implants for orbital reconstruction after resection of spheno-orbital meningioma. *Acta Ophthalmol.* **2019**, 97, 28-29.

156. Genders, S.; Pichardo, S.; van Furth, W.; van den Ende, R.; Notting, I. Custom-made 3D-printed polyetheretherketone (PEEK) implants for orbital reconstruction after resection of benign bony orbital lesions. *Acta Ophthalmol..* **2020**, 98, 8-8.

157. Geng, P.; Zhao, J.; Wu, W. et al. Effects of extrusion speed and printing speed on the 3D printing stability of extruded PEEK filament. *J Manuf. Process.* **2019**, 37, 266-273.

158. Ghomi, E.; Eshkalak, S.; Singh, S.; Chinnappan, A.; Ramakrishna, S.; Narayan, R. Fused filament printing of specialized biomedical devices: a state-of-the art review of technological feasibilities with PEEK. *Rapid Prototyp. J.* **2021**, 27, 592-616.

159. Godlewski, B.; Dominiak, M. Advantages and Disadvantages of the Use of Various Types of Interbody Implants in Cervical Spine Surgery. Critical Review of the Literature. *Orthop. Traumatol. Rehabil.* **2020**, 22, 213-220.

160. Golbang, A.; Harkin-Jones, E.; Wegrzyn, M.; Campbell, G.; Archer, E.; McIlhagger, A. Production and characterization of PEEK/IF-WS2 nanocomposites for additive manufacturing: Simultaneous improvement in processing characteristics and material properties. *Addit. Manuf.* **2020**, 31.

161. Golbang, A.; Mokhtari, M.; Harkin-Jones, E.; Archer, E.; Mcilhagger, A. Additive Manufacturing and Injection Moulding of High-Performance IF-WS2/PEEK Nanocomposites: A Comparative Study. *Front.Mater.* **2021**, 8.

162. Gomez, A.; Leyva, M.; Coutinho, L.; Rosen, E.B.; Kalman, N.; Hobson, M. Preliminary investigation of the dosimetric impact of common dental restorative materials on proton beams. *Med. Phys*. 2021, 48, e553-e554.

163. Gonçalves, J.; Lima, P.; Krause, B. et al. Electrically Conductive Polyetheretherketone Nanocomposite Filaments: From Production to Fused Deposition Modeling. *Polymers (Basel)*. **2018**, 10.

164. Goodall, S.K.; Rampant, P.; Smith, W.; Waterhouse, D.; Rowshanfarzad, P.; Ebert, M.A. Investigation of the effects of spinal surgical implants on radiotherapy dosimetry: A study of 3D printed phantoms. *Med. Phys*. **2021**, 48, 4586-4597.

165. Gopal, S.; Rudrappa, S.; Sekar, A.; Preethish-Kumar, V.; Masapu, D. Customized and Cost-Effective 3D Printed Mold for Cranioplasty: India's First Single Center Experience. *Neurol. India*. **2021**, 69, 611-617.

166. Gordeev, E.; Ananikov, V. Widely accessible 3D printing technologies in chemistry, biochemistry and pharmaceutics: applications, materials and prospects. *Russ. Chem. Rev.* **2020**, 89, 1507-1561.

167. Gordelier, T.; Thies, P.; Turner, L.; Johanning, L. Optimising the FDM additive manufacturing process to achieve maximum tensile strength: a state-of-the-art review. *Rapid Prototyp. J.* **2019**, 25, 953-971.

168. Grajewski, M.; Hermann, M.; Oleschuk, R.D.; Verpoorte, E.; Salentijn, G.I. Leveraging 3D printing to enhance mass spectrometry: A review. *Anal. Chim. Acta*. **2021**, 1166.

169. Grate, J.; Arnquist, I.; Hoppe, E. et al. Mass spectrometric analyses of high performance polymers to assess their radiopurity as ultra low background materials for rare event physics detectors. *Nuclear Instruments & Methods in Physics Research Section A-Accelerators Spectrometers Detectors and Associated Equipment.* **2021**, 985.

170. Grieder, S.; Zhilyaev, I.; Küng, M. et al. Consolidation of Additive Manufactured Continuous Carbon Fiber Reinforced Polyamide 12 Composites and the Development of Process-Related Numerical Simulation Methods. *Polymers (Basel)*. **2022**, 14.

171. Grymak, A.; Aarts, J.M.; Ma, S.; Waddell, J.N.; Choi, J.J.E. Wear Behavior of Occlusal Splint Materials Manufactured By Various Methods: A Systematic Review. *J. Prosthodont*. **2022**, 31, 472-487.

172. Gu, R.D.; Xiao, F.; Wang, L.; Sun, K.J.; Chen, L.L. Biocompatibility of polyetheretherketone for the treatment of orbital bone defects. *Int. J. Ophthalmol*. **2020**, 13, 725-730.

173. Gummadi, S.; Saini, A.; Owusu-Danquah, J.; Sikder, P. Mechanical Properties of 3D-Printed Porous Poly-ether-ether-ketone (PEEK) Orthopedic Scaffolds. *JOM*. **2022**, 74, 3379-3391.

174. Guo, C.; Liu, X.; Liu, G. Surface Finishing of FDM-Fabricated Amorphous Polyetheretherketone and Its Carbon-Fiber-Reinforced Composite by Dry Milling. *Polymers (Basel)*. **2021**, 13.

175. Guo, F.; Huang, S.; Hu, M.; Yang, C.; Li, D.; Liu, C. Biomechanical evaluation of a customized 3D-printed polyetheretherketone condylar prosthesis. *Exp. Ther. Med*. **2021**, 21, 348.

176. Guo, F.; Huang, S.; Liu, N. et al. Evaluation of the mechanical properties and fit of 3D-printed polyetheretherketone removable partial dentures. *Dent. Mater. J*. **2022**.

177. Gupta, S.; Bissoyi, A.; Bit, A. A Review on 3D Printable Techniques for Tissue Engineering. *BioNanoScience*. **2018**, 8, 868-883.

178. Gurchetan, S.; Ranvijay, K.; Rupinder, S.; Mustafizur, R.; Seeram, R. Rheological, mechanical, thermal, tribological and morphological properties of PLA-PEKK-HAp-CS composite. *J. Cent. South Univ.* **2021**, 28, 1615-1626.

179. Guvendiren, M.; Molde, J.; Soares, R.M.D.; Kohn, J. Designing Biomaterials for 3D Printing. *ACS Biomater. Sci. Eng.* **2016**, 2, 1679-1693.

180. Haeri, S.; Wang, Y.; Ghita, O.; Sun, J. Discrete element simulation and experimental study of powder spreading process in additive manufacturing. *Powder Technol.* **2017**, 306, 45-54.

181. Haleem, A.; Javaid, M. Polyether ether ketone (PEEK) and its 3D printed implants applications in medical field: An overview. *Clin. Epidemiology Glob. Health.* **2019**, 7, 571-577.

182. Haleem, A.; Javaid, M. Polyether ether ketone (PEEK) and its manufacturing of customised 3D printed dentistry parts using additive manufacturing. *Clin. Epidemiology Glob.Health.* **2019**, 7, 654-660.

183. Hamsho, R.; Mahardawi, B.; Assi, H.; Alkhatib, H. Polyetheretherketone (PEEK) Implant for the Reconstruction of Severe Destruction in the Maxilla: Case Report. *Plast. Reconstr. Surg. Glob. Open*. **2022**, 10, e4473.

184. Han, X.; Sharma, N.; Spintzyk, S. et al. Tailoring the biologic responses of 3D printed PEEK medical implants by plasma functionalization. *Dent. Mater*. **2022**, 38, 1083-1098.

185. Han, X.; Sharma, N.; Xu, Z. et al. An In Vitro Study of Osteoblast Response on Fused-Filament Fabrication 3D Printed PEEK for Dental and Cranio-Maxillofacial Implants. *J. Clin. Med*. **2019**, 8.

186. Han, X.; Yang, D.; Yang, C. et al. Carbon Fiber Reinforced PEEK Composites Based on 3D-Printing Technology for Orthopedic and Dental Applications. *J. Clin. Med*. **2019**, 8.

187. Harding, M.; Brady, S.; O'Connor, H. et al. 3D printing of PEEK reactors for flow chemistry and continuous chemical processing. *React. Chem. Eng.* **2020**, 5, 728-735.

188. Hassan, M.; Jeon, H.; Kim, G.; Park, K. The effects of infill patterns and infill percentages on energy consumption in fused filament fabrication using CFR-PEEK. *Rapid Prototyp. J.* **2021**, 27, 1886-1899.

189. Hauptmann, N.; Ludolph, J.; Rothe, H. et al. Poly-Alanine-ε-Caprolacton-Methacrylate as Scaffold Material with Tuneable

Biomechanical Properties for Osteochondral Implants. *Int. J. Mol. Sci*. **2022**, 23.

190. He, D.L.; Wang, H.E.; Jiao, Y. et al. 3D printed peek extravascular stent in treatment of nutcracker syndrome in an adolescent boy. *Arch. Dis. Child*. **2021,** 106, A152.

191. He, D.; Liang, J.; Wang, H. et al. 3D-Printed PEEK Extravascular Stent in the Treatment of Nutcracker Syndrome: Imaging Evaluation and Short-Term Clinical Outcome. *Front Bioeng. Biotechnol.* **2020**, 8, 732.

192. He, D.; Wang, H.; Jiao, Y. et al. Successful Resolution of Nutcracker Syndrome with 3D Printed PEEK Extravascular Stent in an Adolescent Boy. *Pediatr. Nephrol.* **2021**, 36, 3407-3407.

193. He, D.; Wang, H.; Jiao, Y. et al. Successful Resolution of Nutcracker Syndrome with 3D Printed PEEK Extravascular Stent in an Adolescent Boy. *Acta Clin Croat*. **2022**, 61, 138-144.

194. Heider, F.C.; Mayer, F.; Mehren, C.; Mayer, H.M. 3-D printed cellular titanium cervical implants enriched with autologous bone marrow leads to a fast, early and solid bony fusion at the cervical spine: A prospective comparison trial with ≥ 1 year follow-up. *Eur. Spine J*. **2018**, 27, 2948.

195. Herford, A.S.; Miller, M.; Lauritano, F.; Cervino, G.; Signorino, F.; Maiorana, C. The use of virtual surgical planning and navigation in the treatment of orbital trauma. *Chin. J. Traumatol*. **2017**, 20, 9-13.

196. Herzmann, M.; Leonhardt, S. Additive manufacturing of patient matched cranial implants in PEEK. *Biomed. Tech*. **2021**, 66, S330.

197. Honigmann, P.; Sharma, N.; Popp, U.; Okolo, B.; Msallem, B.; Thieringer, F. Patient specific surgical implants made of 3D printed PEEK-material, technology and scope of surgical application. *Int. J. Comput. Assisted Radiol. Surg*. **2018**, 13, S215-S216.

198. Honigmann, P.; Sharma, N.; Okolo, B.; Popp, U.; Msallem, B.; Thieringer, FM. Patient-Specific Surgical Implants Made of 3D Printed PEEK: Material, Technology, and Scope of Surgical Application. *Biomed. Res. Int*. **2018**, 4520636.

199. Honigmann, P.; Sharma, N.; Schumacher, R.; Rueegg, J.; Haefeli, M.; Thieringer, F. In-Hospital 3D Printed Scaphoid Prosthesis Using Medical-Grade Polyetheretherketone (PEEK) Biomaterial. *Biomed. Res. Int*. **2021**, 1301028.

200. Hosameldin, A.; Osman, A.; Hussein, M.; Gomaa, A.F.; Abdellatif, M. Three dimensional custom-made PEEK cranioplasty. *Surg. Neurol. Intl*. **2021**, 12.

201. Hsu, C.; Prabhu, G.; Urban, P. Telechemistry 2.0: Remote monitoring of fluorescent chemical reactions. *Hardwarex*. **2021**, 10.

202. Hsu, W.C.; Peng, T.Y.; Kang, C.M.; Chao, F.Y.; Yu, J.H.; Chen, S.F. Evaluating the Effect of Different Polymer and Composite Abutments on the Color Accuracy of Multilayer Pre-Colored Zirconia Polycrystal Dental Prosthesis. *Polymers (Basel)*. **2022**, 14.

203. Hu, B.; Duan, X.; Xing, Z. et al. Improved design of fused deposition modeling equipment for 3D printing of high-performance PEEK parts. *Mech. Mater.* **2019**, 137.

204. Hu, B.; Xing, Z.; Wu, W. et al. Enhancing the mechanical properties of SCF/PEEK composites in FDM via process-parameter optimization. *High Perform. Polym.* **2021**, 33, 914-923.

205. Hu, J.; Zhao, D.; Wang, L. et al. Complication comparison and application improvement of 3D-printed plastic polyether ketone and titanium mesh cranioplasty. *Chin. J. Tissue Eng. Res*. **2022**, 26, 3327-3331.

206. Hu, Q.; Li, W.; Zhang, H.; Liu, D.; Peng, F.; Duan, Y. Research into topology optimization and the FDM method for a space cracked membrane. *Acta Astronaut.* **2017**, 136, 443-449.

207. Hua, Z.; Jun, L.; Guoxiang, C.; Shaoyin, D. Individualized design and clinical application of metacarpophalangeal joint prosthesis: A one-case report. *Chin. J. Tissue Eng. Res*. **2019**, 23, 3164-3169.

208. Huang, G.; Wu, L.; Hu, J. et al. Main Applications and Recent Research Progresses of Additive Manufacturing in Dentistry. *BioMed. Res. Int*. **2022**.

209. Huang, H.; Liu, W.; Liu, Z. An additive manufacturing-based approach for carbon fiber reinforced polymer recycling. *Cirp Ann. Manuf. Technol.* **2020**, 69, 33-36.

210. Hubbe, U.; Beiser, S.; Kuhn, S; et al. A fully ingrowing implant for cranial reconstruction: Results in critical size defects in sheep using 3D-printed titanium scaffold. *Biomater; Adv*. **2022**, 136, 212754.

211. Hudak, R.; Schnitzer, M.; Zivcak, J. IEEE. Additive Manufacturing in Medicine and Tissue Engineering. *2021 IEEE 19th World Symposium on Applied Machine Intelligence and Informatics (SAMI)*. **2021**, 11-12.

212. Hudecki, A.; Wolany, W.; Likus, W. et al. Orbital reconstruction - applied materials, therapeutic agents and clinical problems of restoration of defects. *Eur. J. Pharmacol*. **2021**, 892.

213. Iaccarino, C.; Kolias, A.G.; Roumy, L.G.; Fountas, K.; Adeleye, A.O. Cranioplasty Following Decompressive Craniectomy. *Front. Neurol*. **2020**, 10.

214. Inzana, J.; Trombetta, R.; Schwarz, E.; Kates, S.; Awad, H. 3D Printed Bioceramics for Dual Antibiotic Delivery to Treat Implant-Associated Bone Infection. *Eur. Cell Mater.* **2015**, 30, 232-247.

215. Jafferson, J.; Chatterjee, D. A review on polymeric materials in additive manufacturing. *Materials Today Proceeding*. **2021**, 46, 1349-1365.

216. Javaid, M.; Haleem, A. 3D printing applications towards the required challenge of stem cells printing. *Clin; Epidemiol. Glob. Health.* **2020**, 8, 862-867.

217. Jayaraghul, T.; Karthik, K.; Yaswanth, A.; Venkatesan, M. Nozzle flow characteristics of PEEK (Poly-ether ether ketone) material used in 3D-printing. **2021**, 2963-2967.

218. Jiang, C.; Cheng, Y.; Lin, H.; Chang, Y.; Pasang, T.; Lee, S. Optimization of FDM 3D printing parameters for high strength PEEK using the Taguchi method and experimental validation. *Rapid Prototyp. J.* **2022**, 28, 1260-1271.

219. Jiang, H.; Aihemaiti, P.; Aiyiti, W.; Kasimu, A. Study Of the compression behaviours of 3D-printed PEEK/CFR-PEEK sandwich composite structures. *Virtual Phys. Prototyp.* **2022**, 17, 138-155.

220. Jiang, Q.; Zhang, H.; Rusakov, D.; Yousefi, N.; Bismarck, A. Additive Manufactured Carbon Nanotube/Epoxy Nanocomposites for Heavy-Duty Applications. *ACS Appl. Polymer Mater.* **2021**, 3, 93-97.

221. Jin, H.; Cui, J.; Shao, C. Materials of interbody fusion cage: Advantages and focus of clinical application. *Chin. J. Tissue Eng. Res*. **2022**, 26, 3592-3597.

222. Jindal, S.; Manzoor, F.; Haslam, N.; Mancuso, E. 3D printed composite materials for craniofacial implants: current concepts, challenges and future directions. *International J. Adv. Manufact. Technol.* **2021**, 112, 635-653.

223. Jockusch, J.; Ozcan, M. Additive manufacturing of dental polymers: An overview on processes, materials and applications. *Dent. Mater. J.* **2020**, 39, 345-354.

224. Jung, H.D.; Jang, T.S.; Lee, J.E.; Park, S.J.; Son, Y.; Park, S.H. Enhanced bioactivity of titanium-coated polyetheretherketone implants created by a high-temperature 3D printing process. *Biofabrication*. **2019**, 11, 045014.

225. Kafi, A.; Wu, H.; Langston, J. et al. Evaluation of additively manufactured ultraperformance polymers to use as thermal protection systems for spacecraft. *J. Appl. Polym. Sci.* **2020**, 137.

226. Kang, J.; Wang, L.; Yang, C. et al. Custom design and biomechanical analysis of 3D-printed PEEK rib prostheses. *Biomech. Model Mechanobiol*. **2018**, 17, 1083-1092.

227. Kang, J.; Zhang, J.; Zheng, J.; Wang, L.; Li, D.; Liu, S. 3D-printed PEEK implant for mandibular defects repair - a new method. *J. Mech. Behav. Biomed. Mater*. **2021**, 116, 104335.

228. Karthikeyan, L.; Sudhi, S.; Bhatt, T. et al. Poly(ether ether ketone)s processed through extrusion-machining and 3D printing:

A comparative study on mechanical, thermal and fracture properties at ambient and cryogenic environments. *J. Elastom. Plast.* **2021**, 53, 672-683.

229. Kassem, T.; Sarkar, T.; Nguyen, T.; Saha, D.; Ahsan, F. 3D Printing in Solid Dosage Forms and Organ-on-Chip Applications. *Biosensors*. **2022**, 12.

230. Katschnig, M.; Wallner, J.; Janics, T.; Burgstaller, C.; Zemann, W.; Holzer, C. Biofunctional Glycol-Modified Polyethylene Terephthalate and Thermoplastic Polyurethane Implants by Extrusion-Based Additive Manufacturing for Medical 3D Maxillofacial Defect Reconstruction. *Polymers.* **2020**, 12.

231. Katsifis, G. Radiation dose to tissue inside 3D printed titanium and PEEK scaffolds: Monte Carlo simulation. *Radiother. Oncol.* **2021**, 161, S459-S460.

232. Katsifis, G.; McKenzie, D.R.; Esteves, A.D. et al. 3D printing of bone scaffolds: Prediction of elastic properties and improvement of biological integration. *Australas. Phys. Eng. Sci. Med.* **2019**, 42, 304.

233. Katsifis, G.; McKenzie, D.; Suchowerska, N. Applying the Hashin-Shtrikman bounds to predict stiffness of multicomponent 3D printed structures: Towards regenerative orthopaedic medicine. *J. Comp. Mater.* **2020**, 54, 2173-2183.

234. Katsifis, G.A.; McKenzie, D.R.; Suchowerska, N. Monte Carlo calculations of radiotherapy dose distributions within and around orthopaedic implants. *Phys. Imaging. Radiat. Oncol.* **2022**, 22, 123-130.

235. Katsura, T.; Radwan, A.; Yang, Z.; Nakamura, M.; Nagano, K. Energy conservation using new structured-core and transparent vacuum insulation panels: Numerical simulation with experimental validation. *Solar Energy.* **2019**, 193, 885-905.

236. Kessentini, A.; Mohammed, Sayeed.; Ahmed, G.; Madiouli, J. Design Optimization and FE Analysis of 3D Printed Carbon PEEK Based Mono Leaf Spring. *Micromachines (Basel)*. **2019**, 10.

237. Khudiakova, A.; Berer, M.; Niedermair, S. et al. Systematic analysis of the mechanical anisotropy of fibre-reinforced polymer specimens produced by laser sintering. *Addit. Manufact.* **2020**, 36.

238. Khunt, C.; Makhesana, M.; Mawandiya, B.; Patel, K. Investigations on the influence of printing parameters during processing of biocompatible polymer in Fused Deposition Modelling (FDM). *Adv. Mater.Process. Technol.* **2021**.

239. Kia, C.; Antonacci, C.L.; Wellington, I.; Makanji, H.S.; Esmende, S.M. Spinal Implant Osseointegration and the Role of 3D Printing: An Analysis and Review of the Literature. *Bioengineering (Basel)*. **2022**, 9.

240. Kiapour, A.; Alikhani, P. Effect of implant endplate surface topology on subsidence resistance in cervical interbody fixation: comparison of two 3D printed titanium cervical spine fixation devices. *Spine J*. **2021**, 21, S37-S38.

241. Kim, K.; Noh, H.; Park, K.; Jeon, H.; Lim, S. Characterization of power demand and energy consumption for fused filament fabrication using CFR-PEEK. *Rapid Prototyp. J.* **2022**, 28, 1394-1406.

242. Kim, M.J.; Lee, H.B.; Ha, S.K.; Lim, D.J.; Kim, S.D. Predictive Factors of Surgical Site Infection Following Cranioplasty: A Study Including 3D Printed Implants. *Front. Neurol*. **2021**, 12, 745575.

243. Kinsman, M.; Aljuboori, Z.; Ball, T.; Nauta, H.; Boakye, M. Rapid high-fidelity contour shaping of titanium mesh implants for cranioplasty defects using patient-specific molds created with low-cost 3D printing: A case series. *Surg. Neurol. Int*. **2020**, 11, 288.

244. Koller, M.; Rafter, D.; Shok, G.; Murphy, S.; Kiaei, S.; Samadani, U. A retrospective descriptive study of cranioplasty failure rates and contributing factors in novel 3D printed calcium phosphate implants compared to traditional materials. *3D Print. Med.* **2020**, 6.

245. Koper, D.; ter Laak-Poort, M.; Lethaus, B. et al. Cranioplasty with patient-specific implants in repeatedly reconstructed cases. *J. Cranio Maxillofac. Surg.* **2019**, 47, 709-714.

246. Krafft, P.R.; Osburn, B.; Vivas, A.C.; Rao, G.; Alikhani, P. Novel Titanium Cages for Minimally Invasive Lateral Lumbar

Interbody Fusion: First Assessment of Subsidence. *Spine Surg. Relat. Res*. **2020**, 4, 171-177.

247. Kravchuk, A.D.; Potapov, A.A.; Panchenko, V.Y. et al. Additive technologies in neurosurgery. *Zh Vopr Neirokhir Im N N Burdenko*. **2018**, 82, 97-104.

248. Kreß, S.; Schaller-Ammann, R.; Feiel, J. et al. Innovative Platform for the Advanced Online Monitoring of Three-Dimensional Cells and Tissue Cultures. *Cells*. **2022**, 11.

249. Kroczek, K.; Turek, P.; Mazur, D. et al. Characterisation of Selected Materials in Medical Applications. *Polymers*. **2022**, 14.

250. Kruse, H.; Lewin, W.; Suchowerska, N. et al. Plasma immersion ion-implanted 3D-printed PEEK bone implants: In vivo sheep study shows strong osseointegration. *Plasma Process. Polym*. **2022**, 19.

251. Kruse, H.; McKenzie, D.; Clark, J.; Suchowerska, N. Plasma ion implantation of 3D-printed PEEK creates optimal host conditions for bone ongrowth and mineralisation. *Plasma Process. Polym*. **2021**, 18.

252. Kudla-Hooper, M.; Batchelar, D.; Moore, J.; Crook, J.; Bachand, F. Development and Design of Custom 3D Printed Cylindrical Templates for Interstitial Gynecological Brachytherapy. *Radiother. Oncol*. **2021**, 163, S36.

253. Kumar, M.; Khan, M.; Mishra, S. Usage of Poly-Ether-Ether-Ketone Polymer for the Biomedical Application-A Critical Review. *In: Praveen Kumar, A., Dirgantara, T., Krishna, P.V. (eds) Advances in Lightweight Materials and Structures . Springer Proceedings in Materials, vol 8. Springer, Singapore.* **2020**, 371-379.

254. Kumar, N. Current trends and future scope in 3D printing for surgical management of spine pathologies. *Global Spine J*. **2022**, 12, 305S.

255. Kusoglu, I.M.; Doñate-Buendía, C.; Barcikowski, S.; Gökce, B. Laser Powder Bed Fusion of Polymers: Quantitative Research Direction Indices. *Materials (Basel)*. **2021**, 14.

256. Kwarcinski, J.; Boughton, P.; van Gelder, J.; Damodaran, O.; Doolan, A.; Ruys, A. Clinical evaluation of rapid3Dprint-formedimplants for surgical reconstruction of large cranial defects. *Anz J. Surg*. **2021**, 91, 1226-1232.

257. Kwarcinski, J.; Boughton, P.; Ruys, A.; van Gelder, J. The rapid templating process for large cranial defects. *Neuromethods*. **2018**, 139, 329-348.

258. Lai, W.; Wang, Y.; Fu, H.; He, J. Hydroxyapatite/polyetheretherketone nanocomposites for selective laser sintering: Thermal and mechanical performances. *E-Polymers*. **2020**, 20, 542-549.

259. Lal, B.; Ghosh, M.; Agarwal, B.; Gupta, D.; Roychoudhury, A. A novel economically viable solution for 3D printing-assisted cranioplast fabrication. *Br. J. Neurosurg*. **2020**, 34, 280-283.

260. Laratta, J.L.; Vivace, B.J.; López-Peña, M. et al. 3D-printed titanium cages without bone graft outperform PEEK cages with autograft in an animal model. *Spine J*. **2022**, 22, 1016-1027.

261. Larochelle, R.D.; Mann, S.E.; Ifantides, C. 3D Printing in Eye Care. *Ophthalmol. Ther*. **2021**, 10, 733-752.

262. Lau, N.; Tsai, M.; Chen, D.; Chen, C.; Cheng, K. Preparation and Characterization for Antibacterial Activities of 3D Printing Polyetheretherketone Disks Coated with Various Ratios of Ampicillin and Vancomycin Salts. *Appl. Sci*. **2020** , 10.

263. Lau, N.C.; Lai, Y.C.; Chen, D.W. ; Cheng, K.W. Antibacterial Activity Studies of 3D-Printing Polyetheretherketone Substrates with Surface Growth of 2D TiO(2)/ZnO Rodlike Arrays. *ACS Omega*. **2022**,7, 9559-9572.

264. Laubach, M.; Kobbe, P.; Hutmacher, D.W. Biodegradable interbody cages for lumbar spine fusion: Current concepts and future directions. *Biomaterials*. **2022**, 288, 121699.

265. Lawand, G.; Ismail, Y.; Revilla-León, M.; Tohme, H. Effect of implant scan body geometric modifications on the trueness and scanning time of complete arch intraoral implant digital scans: An in vitro study. *J. Prosthet. Dent*. **2022**, S0022-3913, 00378-X.

266. Lawson, Z.T.; Han, J.; Saunders, W.B.; Grunlan, M.A.; Moreno, M.R.; Robbins, A.B. Methodology for performing biomechanical push-out tests for evaluating the osseointegration of calvarial defect repair in small animal models. *MethodsX*. **2021**, 8, 101541.

267. Lee, C.; Vandenbrande, J.; Goetz, A.; Ganter, M.; Storti, D.; Boydston, A. Room temperature extrusion 3D printing of polyether ether ketone using a stimuli-responsive binder. *Addit. Manufact.* **2019**, 28, 430-438.

268. Lee, S.; Cho, S.; Kim, W. Development of thermo-fluid simulation technique for extruder and chamber of FDM-type 3D printer for printing high-melting-point materials. *Microsystem Technol.* **2022**.

269. Lee, W.T.; Chen, Y.C. Digitally Fabricated Dentures for Full Mouth Rehabilitation with Zirconia, Polyetheretherketone and Selective Laser Melted Ti-6Al-4V Material. *Int. J. Environ. Res. Public Health*. **2022**, 19.

270. Lehner, L.; Jakab, C.; Czeibert, K. Cranioplasty with a digitally designed and custom-made implant in a dog. *Magyar Allatorvosok Lapja.* **2022**, 144, 259-268.

271. Lei, M.; Hamel, C.; Chen, K. et al. Thermomechanical behaviors of polyether ether ketone (PEEK) with stretch-induced anisotropy. *J. Mechan. Phys Solids. 2021*, 148.

272. Leiser, Y. Patient specific implants in craniofacial reconstruction of trauma patients. *Int. J. Oral Maxillofac. Surg*. **2015**, 44, e97-e98.

273. Len'shina, N.; Shurygina, M.; Chesnokov, S. Photoreduction Reaction of Carbonyl-Containing Compounds in the Synthesis and Modification of Polymers. *Polym. Sci. B.* **2021**, 63, 657-690.

274. Leonhardt, S.; Haerst, M.; Fischer, S.; Pammer, S.; Bauer, F.; Eblenkamp, M. "Medical Grade" polymeric additive manufacturing technologies. *Biomed. Tech*. **2017**, 62, S458.

275. Levitsky, Y.; Pegouske, D.J.; Hammer, S.S. et al. Micro-respirometry of whole cells and isolated mitochondria. *RSC Adv*. **2019**, 9, 33257-33267.

276. Li, J.; Durandet, Y.; Huang, X.; Sun, G.; Ruan, D. Additively manufactured fiber-reinforced composites: A review of mechanical behavior and opportunities. *J. Mater Sci. Technol.* **2022**, 119, 219-244.

277. Li, L.; Gao, H.; Wang, C.; Ji, P.; Huang, Y.; Wang, C. Assessment of Customized Alveolar Bone Augmentation Using Titanium Scaffolds vs Polyetheretherketone (PEEK) Scaffolds: A Comparative Study Based on 3D Printing Technology. *ACS Biomater. Sci Eng*. **2022**, 8, 2028-2039.

278. Li, M.; Tian, F.; Jiang, J. et al. Robust and Multifunctional Porous Polyetheretherketone Fiber Fabricated via a Microextrusion CO(2) Foaming. *Macromol. Rapid Commun*. **2021**, 42, e2100463.

279. Li, M.; Zhou, M.; Jiang, J.; Tian, F.; Gao, N.; Zhai, W. Microextrusion Foaming of Polyetheretherketone Fiber: Mechanical and Thermal Insulation Properties. *Adv. Eng. Mater.* **2022**, 24.

280. Li, P.; Jiang, W.; Yan, J. et al. A novel 3D printed cage with microporous structure and in vivo fusion function. *J. Biomed. Mater. Res A*. **2019**, 107, 1386-1392.

281. Li, Q.; Wu, T.; Zhao, W.; Ji, J.; Wang, G. Laser-Induced Corrugated Graphene Films for Integrated Multimodal Sensors. *ACS Appl. Mater. Interfaces*. **2021**, 13, 37433-37444.

282. Li, Q.; Zhao, W.; Li, Y.; Yang, W.; Wang, G. Flexural Properties and Fracture Behavior of CF/PEEK in Orthogonal Building Orientation by FDM: Microstructure and Mechanism. *Polymers (Basel)*. **2019**, 11.

283. Li, Q.; Wu, T.; Zhao, W.; Li, Y.; Ji, J.; Wang, G. 3D printing stretchable core-shell laser scribed graphene conductive network for self-powered wearable devices. *Comp.B Eng*. **2022**, 240.

284. Li, Q.; Zhao, W.; Niu, B. et al. 3D printing high interfacial bonding polyether ether ketone components via pyrolysis reactions. *Mater. Des.* **2021**, 198.

285. Li, S.; Wang, T.; Hu, J. et al. Surface porous poly-ether-ether-ketone based on three-dimensional printing for load-bearing orthopedic implant. *J. Mech. Behav. Biomed Mater*. **2021**, 120, 104561.

286. Li, W.; Sang, L.; Jian, X.; Wang, J. Influence of sanding and plasma treatment on shear bond strength of 3D-printed PEI, PEEK and PEEK/CF. *Int. J. Adhes. Adhes.* **2020**, 100.

287. Li, W.; Wang, J.; Sang, L. et al. Effect of IR-laser treatment parameters on surface structure, roughness, wettability and bonding properties of fused deposition modeling-printed PEEK/CF. *J. Appl. Polym. Sci.* **2021**, 138.

288. Li, W.; Zheng, C.; Zhang, Y. PEEK-Barium sulfate composite for three-dimensional virtual reconstruction of a printed human in vitro model using CT. *Rapid Prototyp. J.* **2022**, 28, 1-9.

289. Li, X.; Yu, D.; Cao, T. et al. 3D printing and dynamic modeling of a polymer-based bimodal piezoelectric motor. *Smart Mater. Struct.* **2021**, 30.

290. Li, Y.; Lou, Y. Tensile and Bending Strength Improvements in PEEK Parts Using Fused Deposition Modelling 3D Printing Considering Multi-Factor Coupling. *Polymers (Basel)*. **2020**, 12.

291. Li, Y.; Li, Z.; Tian, L. et al. Clinical application of 3D-printed PEEK implants for repairing mandibular defects. *J. Craniomaxillofac. Surg*. **2022**, 50, 621-626.

292. Liao, C.; Wu, P.; Lee, C. Customized PEEK Implants with Microporous and Surface Modification Using 3D Printing. *Proceedings of the ASME 2019 International Design Engineering Technical Conferences and Computers and Information in Engineering Conference. Volume 4: 24th Design for Manufacturing and the Life Cycle Conference; 13th International Conference on Micro- and Nanosystems*. Anaheim, California, USA. August 18–21, 2019. V004T08A013. ASME. **2020**.

293. Liaw, C.Y.; Tolbert, J.W.; Chow, L.W.; Guvendiren, M. Interlayer bonding strength of 3D printed PEEK specimens. *Soft Matter*. **2021**, 17, 4775-4789.

294. Lim, D.; Renteria, E.S.; Sime, D.S. et al. Bioreactor design and validation for manufacturing strategies in tissue engineering. *Bio-Des. Manufact*. **2022**, 5, 43-63.

295. Lim, K.; Park, T.; Lee, S.; Park, S. Design and Biomechanical Verification of Additive Manufactured Composite Spinal Cage Composed of Porous Titanium Cover and PEEK Body. *Appl. Sci.* **2019**, 9.

296. Limaye, N.; Veschini, L.; Coward, T. 390 Assessing Biocompatibility & Mechanical Testing of 3D-Printed PEEK Versus Milled PEEK in Maxillofacial Surgical Applications. *Br. J. Surg.* **2022**, 109.

297. Lin, L.; Zhou, J. Application status of 3D printed polyetheretherketone and its composite in bone defect repair. *Chin. J. Tissue Eng. Res*. **2020**, 24, 1622-1628.

298. Lin, L.; Ecke, N.; Huang, M.; Pei, X.; Schlarb, A. Impact of nanosilica on the friction and wear of a PEEK/CF composite coating manufactured by fused deposition modeling (FDM). *Comp. B Eng*. **2019**, 177.

299. Linder, L.K.B.; Birgersson, U.; Engqvist, H.; Thomsen, P.; Engstrand, T. In-situ bone regeneration of cranial defects using synthetic ceramic implant. *J. Neurotrauma*. **2018**, 35, A79-A80.

300. Ling, Q.; He, E.; Zhang, H.; Lin, H.; Huang, W. A novel narrow surface cage for full endoscopic oblique lateral lumbar interbody fusion: A finite element study. *J. Orthop. Sci*. **2019**, 24, 991-998.

301. Litowczenko, J.; Woźniak-Budych, M.J.; Staszak, K.; Wieszczycka, K.; Jurga, S.; Tylkowski, B. Milestones and current achievements in development of multifunctional bioscaffolds for medical application. *Bioact. Mater.* **2021**, 6, 2412-2438.

302. Littlefield, A.; Bartolucci, S.; Maurer, J. A Study on the Use of Graphene-PEEK Composites as High Temperature Adhesives Mechanical Properties and Microwave Activation. Proceedings of the ASME 2017 International Mechanical Engineering Congress and Exposition. Volume 14: Emerging Technologies; Materials: Genetics to Structures; Safety Engineering and Risk Analysis. *Tampa, Florida, USA. November 3–9, 2017. V014T11A041. ASME.* **2018**.

303. Liu, C.; Hsieh, Y.; Sun, T. et al. Design and Test of Additive Manufacturing for Coating Thermoplastic PEEK Material. *2016 IEEE International Conference on Industrial Technology (ICIT).* **2016**, 1158-1162.

304. Liu, D.; Fu, J.; Fan, H. et al. Application of 3D-printed PEEK scapula prosthesis in the treatment of scapular benign fibrous histiocytoma: A case report. *J. Bone Oncol*. **2018**, 12, 78-82.

305. Liu, F.; Kang, H.; Liu, Z. et al. 3D Printed Multi-Functional Scaffolds Based on Poly(epsilon-Caprolactone) and Hydroxyapatite Composites. *Nanomaterials*. **2021**,11.

306. Liu, H.; Cheng, X.; Yang, X.; Zheng, G.; Guo, Q. Experimental study on parameters of 3D printing process for PEEK materials. *IOP Conf. Ser.: Mater. Sci. Eng.* **2019**.

307. Liu, W.; Huang, H.; Zhu, L.; Liu, Z. Integrating carbon fiber reclamation and additive manufacturing for. *Comp. B Eng.* **2021**, 215.

308. Liu, X.; Huang, L.; Zhang, H. et al. Facile Amidogen Bio-Activation Method Can Boost the Soft Tissue Integration on 3D Printed Poly-Ether-Ether-Ketone Interface. *Adv.Mater. Interfaces*. **2021**, 8.

309. Liu, Y.; Davies, R.; Yi, N.; McCutchion, P.; Chen, B.; Ghita, O. Multiscale Porous Poly (Ether-Ether-Ketone) Structures Manufactured by Powder Bed Fusion Process. *3D Print. Addit. Manuf.* **2022**.

310. Liu, Y.; Yi, N.; Davies, R.; McCutchion, P.; Ghita, O. Powder Bed Fusion Versus Material Extrusion: A Comparative Case Study on Polyether-Ether-Ketone Cranial Implants. *3D Print. Addit. Manuf.* **2022**.

311. Liu, Y.; Rath, B.; Tingart, M.; Eschweiler, J. Role of implants surface modification in osseointegration: A systematic review. *J. Biomed. Mater. Res. Part A*. **2020**, 108, 470-484.

312. Liu, Y.; Zhai, X.; Deng, Y.; Wu, D. Tribological Property of Selective Laser Melting-Processed 316L Stainless Steel against Filled PEEK under Water Lubrication. *Tribol. Trans.* **2019**, 62, 962-970.

313. Liu, Z.; Wang, Y.; Zhang, Y.; Ming, Y.; Sun, Z.; Sun, H. Application of 3d printed interbody fusion cage for cervical spondylosis of spinal cord type: Half-year follow-up of recovery of cervical curvature and intervertebral height. *Chin. J. Tissue Eng. Res*. **2020**, 25, 849-853.

314. Liu, Z.; Wang, G.; Huo, Y.; Zhao, W. Research on Precise Control of 3D Print Nozzle Temperature in PEEK Material. *AIP Conference Proceedings.* **2017,** 1890, 040076.

315. Liu, Z.; Zhang, M.; Wang, Z. et al. 3D-printed porous PEEK scaffold combined with CSMA/POSS bioactive surface: A strategy for enhancing osseointegration of PEEK implants. *Comp. B Eng.* **2022**, 230.

316. Livingstone, I.; Bastawrous, A.; Giardini, M.E.; Jordan, S. Peek: Portable eye examination kit. the smartphone ophthalmoscope. *Invest. Ophthalmol. Vis. Sci*. **2014**, 55, 1612.

317. Lommen, J.; Schorn, L.; Sproll, C. et al. Reduction of CT Artifacts Using Polyetheretherketone (PEEK), Polyetherketoneketone (PEKK), Polyphenylsulfone (PPSU), and Polyethylene (PE) Reconstruction Plates in Oral Oncology. *J. Oral Maxillofac. Surg*. **2022**, 80, 1272-1283.

318. Lough, C.; Bezold, W.; Feltgz, K.; Middleton, K.; Skelley, N.; Gieg, S. Mechanical comparison of 3d-printed plates and screws for open reduction and internal fixation of fractures. *Orthop. J. Sports Med*. **2020**, 8.

319. Lu, L.; Chen, X.Y.; Li, Y.P.; Gang, L.; Tu, Y. Research status of skull repair materials and the prospect of three-dimensional printing technology. *Chin. J. Tissue Eng. Res*. **2016**, 20, 7885-7890.

320. Luengo, F.; Sanz-Esporrín, J.; Noguerol, F.; Sanz-Martín, I.; Sanz-Sánchez, I.; Sanz, M. In vitro effect of different implant decontamination methods in three intraosseous defect configurations. *Clin. Oral Implants Res*. **2022**.

321. Luo, M.; Huang, S.; Man, Z.; Cairney, J.; Chang, L. Tribological behaviour of fused deposition modelling printed short carbon fibre reinforced nylon composites with surface textures under dry and water lubricated conditions. *Friction*. **2022**.

322. Luo, M.; Tian, X.; Shang, J.; Zhu, W.; Li, D.; Qin, Y. Impregnation and interlayer bonding behaviours of 3D-printed continuous carbon-fiber-reinforced poly-ether-ether-ketone composites. *Comp. A Appl. Sci. Manuf.* **2019**, 121, 130-138.

323. Luo, M.; Tian, X.; Shang, J. et al. Bi-scale interfacial bond behaviors of CCF/PEEK composites by plasma-laser cooperatively assisted 3D printing process. *Comp. A Appl. Sci. Manufact*. **2020**, 131.

324. Luo, M.; Tian, X.; Zhu, W.; Li, D. Controllable interlayer shear strength and crystallinity of PEEK components by laser-assisted material extrusion. *J. Mater. Res.* **2018**, 33, 1632-1641.

325. Lv, Y.; Thomas, W.; Chalk, R.; Singamneni, S. Flame retardant polymeric materials for additive manufacturing. *Mater. Today Proceed*. **2020**, 33, 5720-5724.

326. Younis, M.; Unkovskiy, A.; Drexler, T.; Qian, J.; Wan, G.; Spintzyk, S. The impact of non-thermal plasma on the adhesion of polyetherketoneketone (PEKK) to a veneering composite system. *J Mech Behav Biomed Mater*. **2020**, 112.

327. Ma, L.; Ke, W.; Liao, Z. et al. Small extracellular vesicles with nanomorphology memory promote osteogenesis. *Bioact. Mater*. **2022**, 17, 425-438.

328. Ma, L.; Li, G.; Lei, J. et al. Nanotopography Sequentially Mediates Human Mesenchymal Stem Cell-Derived Small Extracellular Vesicles for Enhancing Osteogenesis. *ACS Nano*. **2021**.

329. Ma, X.Y.; Wang, D. Open fracture repair: Wound infection, implant selection and evaluation of prognosis. *Chin. J. Tissue Eng. Res*. **2015**, 19, 4258-4264.

330. Maglaras, C.; Maglaras, V. ; Stevens, A.V. Static mechanical properties of 3D printed trans-modular PLA scaffold for bone grafting applications. *J. Orthop. Res*. **2016**, 34.

331. Mallya, P.; Juneja, M. Rapid prototyping of orthopedic implant materials for cranio-facial reconstruction: A Survey. *Mater. Today Proceed*. **2021**, 5207-5213.

332. Mangano, F.G.; Marchiori, F.; Mangano, C.; Admakin, O. Solid index and reverse implant library for the fabrication of a bar for overdenture: a proof of concept. *Int. J. Comput. Dent*. **2021,** 24, 331-343.

333. Mangano, F.; Mangano, C.; Margiani, B.; Admakin, O. Combining Intraoral and Face Scans for the Design and Fabrication of Computer-Assisted Design/Computer-Assisted Manufacturing (CAD/CAM) Polyether-Ether-Ketone (PEEK) Implant-Supported Bars for Maxillary Overdentures. *Scanning.* **2019**, 4274715.

334. Mansour, S.; Delaune, A.; Manneveau, M. et al. Soft and effective detoxification of a VX simulant in a nylon 3D printed basic flow reactor. *Green Chem.* **2021**, 23, 7522-7527.

335. Manzoor, F.; Golbang, A.; McIlhagger, A.; Harkin-Jones, E.; Crawford, D.; Mancuso, E. Effect of Zn-nanoHA concentration on the mechanical performance and bioactivity of 3D printed PEEK composites for craniofacial implants. *Plastics Rubber and Composites.* **2022**.

336. Manzoor, F.; Golbang, A.; Dixon, D. et al. 3D Printed Strontium and Zinc Doped Hydroxyapatite Loaded PEEK for Craniomaxillofacial Implants. *Polymers (Basel)*. **2022**, 14.

337. Manzoor, F.; Golbang, A.; Jindal, S. et al. 3D printed PEEK/HA composites for bone tissue engineering applications: Effect of material formulation on mechanical performance and bioactive potential. *J. Mech. Behav. Biomed. Mater*. **2021**, 121, 104601.

338. Marcian, P.; Narra, N.; Borak, L.; Chamrad, J.; Wolff, J. Biomechanical performance of cranial implants with different thicknesses and material properties: A finite element study. *Comput. Biol. Med.* **2019**, 109, 43-52.

339. Martinez, F.; Alister, J.; Uribe, F.; Olate, S. Late Correction of Orbital Deformities Using Customized 3D Implant. *Int. J. Morphol.* **2021**, 39, 1683-1687.

340. Masia, J.; Pamias, J.; Gutiérrez, J.; Saez, M.; Bescós, C. New generation of maxillomandibular reconstruction with free fibrula flap using computer-aided design/computer-aided manufacturing solutions. *Int. J. Comput. Assisted Radiol. Surg*. **2017**, 12,

S171-S172.

341. Matschinski, A.; Ziegler, P.; Abstreiter, T.; Wolf, T.; Drechsler, K. Fiber Formation of Printed Carbon Fiber/Poly (Ether Ether Ketone) with Different Nozzle Shapes. *Polym. Int.* **2021**, 70, 1109-1117.

342. Mau, R.; Jüttner, G.; Gao, Z. et al. Micro injection molding of individualized implants using 3D printed molds manufactured via digital light processing. *Biomed. Tech*. **2021**, 66, S308.

343. Maydanshahi, M.R.; Nazarian, A.; Eygendaal, D.; Ebrahimzadeh, M.H.; Kachooei, A.R.; Mousavi Shaegh, S.A. 3D printing-assisted fabrication of a patient-specific antibacterial radial head prosthesis with high periprosthetic bone preservation. *Biomed. Mater*. **2021**, 16.

344. Mayer, F.; Heider, F.; Haasters, F.; Mehren, C. Radiological and Clinical Outcomes after Anterior Cervical Discectomy and Fusion (ACDF) with an Innovative 3D Printed Cellular Titanium Cage Filled with Vertebral Bone Marrow. *Biomed. Res. Int*. **2022**, 6339910.

345. McAllister, C.; Navani, D. JoVE Monthly Highlights: March 2018. *JoVE.* **2018**.

346. McCarthy, C.; Fenlon, M.; Nibali, L.; Addison, O. Analysis of curved titanium surfaces following ultrasonic instrumentation to manage peri-implant mucositis - an in-vitro study. *J. Clin. Periodontol*. **2022**, 49, 72.

347. McDonnell, J.M.; Nagassima Rodrigues Dos Reis, K.; Ahern, D.P, Mahon, J.; Butler, J.S. Are Carbon-fiber Implants More Efficacious Than Traditional Metallic Implants for Spine Tumor Surgery? *Clin. Spine Surg*. **2021**, 34, 159-162.

348. McGilvray, K.; Seim, H.; Berven, S. et al. Biomechanical and histologic comparison of a novel 3-D printed titanium interbody cage to standard peek cages. *J. Orthop. Res*. **2017**, 35.

349. McGilvray, K.C.; Puttlitz, C.M.; Berven, S.H. et al. Biomechanical and histologic comparison of a novel 3-d printed porous titanium interbody cage to peek. *Spine J*. **2016**, 16, S363-S364.

350. McGilvray, K.C.; Easley, J.; Seim, H.B. et al. Bony ingrowth potential of 3D-printed porous titanium alloy: a direct comparison of interbody cage materials in an in vivo ovine lumbar fusion model. *Spine J*. **2018**, 18, 1250-1260.

351. Meftahi, M.; Fields, E.; Song, W. The Design of a Novel Direction Modulated Brachytherapy Vaginal Cylinder Using GEANT4 Monte Carlo Simulation Code. *Brachytherapy*. **2021**, 20, S10-S11.

352. Meisel, H.J.; Agarwal, N. Commentary on "biomaterials in spinal implants: A review." *Neurospine*. **2020**, 17, 111-113.

353. Memarian, P.; Pishavar, E.; Zanotti, F. et al. Active Materials for 3D Printing in Small Animals: Current Modalities and Future Directions for Orthopedic Applications. *Int. J. Mol. Sci*. **2022**, 23.

354. Mendaza-DeCal, R.; Peso-Fernandez, S.; Rodriguez-Quiros, J. Test of Designing and Manufacturing a Polyether Ether Ketone Endoprosthesis for Canine Extremities by 3D Printing. *Front. Mech. Eng*. **2021**, 7.

355. Mendaza-DeCal, R.; Ballesteros, Y.; Peso-Fernandez, S.; Paz, E.; Del Real-Romero, J.C.; Rodriguez-Quiros, J. Biomechanical Test of a New Endoprosthesis for Cylindrical Medullary Canals in Dogs. *Front. Vet. Sci*. **2022**, 9, 887676.

356. Menzel, F.; Klein, T.; Ziegler, T.; Neumaier; J. 3D-printed PEEK reactors and development of a complete continuous flow system for chemical synthesis. *React. Chem. Eng*. **2020**, 5, 1300-1310.

357. Mian, S.H.; Moiduddin, K.; Elseufy, S.M.; Alkhalefah, H. Adaptive Mechanism for Designing a Personalized Cranial Implant and Its 3D Printing Using PEEK. *Polymers (Basel)*. **2022**, 14.

358. Miller, A.T.; Safranski, D.L.; Wood, C.; Guldberg, R.E.; Gall, K. Deformation and fatigue of tough 3D printed elastomer scaffolds processed by fused deposition modeling and continuous liquid interface production. *J. Mech. Behav. Biomed. Mater*. **2017**, 75, 1-13.

359. Minguez, J.P.; Laratta, J.L.; Gum, J.L.; Pugely, A.J.; Glassman, S.D. 63. Three-dimensional printing of titanium without bone

graft outperforms PEEK + autologous iliac crest bone graft in sheep interbody fusion model. *Spine J.* **2020**, 20, S30.

360. Miri, S.; Kalman, J.; Canart, J.; Spangler, J.; Fayazbakhsh, K. Tensile and thermal properties of low-melt poly aryl ether ketone reinforced with continuous carbon fiber manufactured by robotic 3D printing. *Int. J. Adv. Manuf. Technol.* **2022**.

361. Mohamed, T.; Barhoumi, N.; Lamnawar, K.; Maazouz, A.; Znaidi, A. Optimization of fused deposition modeling process parameters using the Taguchi method to improve the tensile properties of 3D-printed polyether ether ketone. *Proceedings of the Institution of Mechanical Engineers Part L-Journal of Materials-Design and Applications.* **2021**, 235, 2565-2573.

362. Mohammad, K. Customised Cranioplasty Implant for Decompressive Craniectomy Patients ? A Technical Note. *Turk. Neurosurg*. **2019**, 29, 148-150.

363. Mrówka, M.; Machoczek, T.; Jureczko, P. et al. Mechanical, Chemical, and Processing Properties of Specimens Manufactured from Poly-Ether-Ether-Ketone (PEEK) Using 3D Printing. *Materials (Basel)*. **2021**, 14.

364. Mueller, K.; Pammer, S.; Leonhardt, S.; Mela, P. A Feature-Specific Local Cooling System to Control Tensile Strength and Dimensional Accuracy in Fused Filament Fabrication. *3D Print. Addit. Manuf.* **2021**.

365. Muthiah, N.; Yolcu, Y.U.; Alan, N.; Agarwal, N.; Hamilton, D.K.; Ozpinar, A. Evolution of polyetheretherketone (PEEK) and titanium interbody devices for spinal procedures: a comprehensive review of the literature. *Eur. Spine J.* **2022**.

366. Mutyala, R.; Park, K.; Gunay, E. et al. Effect of FFF process parameters on mechanical strength of CFR-PEEK outputs. *Int. J. Interact.* **2022**.

367. Nahumi, N.; Shohet, M.R.; Bederson, J.B.; Elahi, E. Frontorbital Fibrous Dysplasia Resection and Reconstruction With Custom Polyetherlatone Alloplast. *J. Craniofac. Surg*. **2015**, 26, e720-722.

368. Nakonieczny, D.; Antonowicz, M.; Heim, T. et al. Cenospheres-Reinforced PA-12 Composite: Preparation, Physicochemical Properties, and Soaking Tests. *Polymers.* **2022**, 14.

369. Nassar, H.I.; Abdelaziz, M.S. Retention of bar clip attachment for mandibular implant overdenture. *BMC Oral Health*. **2022**, 22, 227.

370. Neff, C.; Elston, E.; Burfeindt, M.; Crane, N.; Schrand, A. A fundamental study of printed ink resiliency for harsh mechanical and thermal environmental applications. *Addit. Manuf.* **2018**, 20, 156-163.

371. Neff, C.; Elston, E.; Schrand, A.; Crane, N. Adhesion testing of printed inks while varying the surface treatment of polymer substrates. *J. Adhes.* **2021**, 97, 399-416.

372. Negm, E.E.; Aboutaleb, F.A.; Alam-Eldein, A.M. Virtual Evaluation of the Accuracy of Fit and Trueness in Maxillary Poly(etheretherketone) Removable Partial Denture Frameworks Fabricated by Direct and Indirect CAD/CAM Techniques. *J. Prosthodont*. **2019**, 28, 804-810.

373. Ng, M.; Brennan, S. Mechanical performance analysis of ULTEM 9.85 in a heated, irradiated environment. ASME 2018 International Mechanical Engineering Congress and Exposition, November 9–15, 2018, Pittsburgh, Pennsylvania, USA. **2019,** 9.

374. Ntilikina, Y.; Charles, Y.P.; Persohn, S.; Skalli, W. Influence of double rods and interbody cages on quasistatic range of motion of the spine after lumbopelvic instrumentation. *Eur. Spine J.* **2020**, 29, 2980-2989.

375. Nulty, J.; Freeman, F.E.; Browe, D.C. et al. 3D bioprinting of prevascularised implants for the repair of critically-sized bone defects. *Acta Biomater*. **2021**, 126, 154-169.

376. Nyman, L.; Kestila, A.; Porri, P. et al. Constructing Spacecraft Components Using Additive Manufacturing and Atomic Layer Deposition: First Steps for Integrated Electric Circuitry. *J. Aerospace Eng.* **2021**, 34.

377. O'Leary, J.; Lee, K.; Peek, N. A Grammar of Digital Fabrication Machines. **2021**.

378. Oh, J.H. Recent advances in the reconstruction of cranio-maxillofacial defects using computer-aided design/computer-aided manufacturing. *Maxillofac. Plast. Reconstr. Surg.* **2018**, 40, 2.

379. Oladapo, B.I.; Ismail, S.O.; Bowoto, O.K.; Omigbodun, F.T.; Olawumi, M.A.; Muhammad, M.A. Lattice design and 3D-printing of PEEK with Ca(10)(OH)(PO(4))(3) and in-vitro bio-composite for bone implant. *Int. J. Biol. Macromol.* **2020**, 165, 50-62.

380. Oladapo, B.I.; Ismail, S.O.; Ikumapayi, O.M.; Karagiannidis, P.G. Impact of rGO-coated PEEK and lattice on bone implant. *Colloids Surf. B Biointerfaces*. **2022**, 216, 112583.

381. Oladapo, B.I.; Zahedi, S.A.; Ismail, S.O.; Omigbodun, F.T. 3D printing of PEEK and its composite to increase biointerfaces as a biomedical material- A review. *Colloids Surf. B Biointerfaces*. **2021**, 203, 111726.

382. Oladapo, B.; Ismail, S.; Adebiyi, A.; Omigbodun, F.; Olawumi, M.; Olawade, D. Nanostructural interface and strength of polymer composite scaffolds applied to intervertebral bone. *Colloids Surf. A Physicochem. Eng.* **2021**, 627.

383. Oladapo, B.; Obisesan, O.; Oluwole, B.; Adebiyi, V.; Usman, H.; Khan, A. Mechanical characterization of a polymeric scaffold for bone implant. *J. Mater. Sci.* **2020**, 55, 9057-9069.

384. Oladapo, B.; Zahedi, S. Improving bioactivity and strength of PEEK composite polymer for bone application. *Mater. Chem. Phys.* **2021**, 266.

385. Oladapo, B.; Zahedi, S.; Chong, S.; Omigbodun, F.; Malachi, I. RETRACTION: 3D printing of surface characterization and finite element analysis improvement of PEEK-HAP-GO in bone implant. *Int. J. Adv. Manuf. Technol.* **2021**, 115, 3361-3361.

386. Oladapo, B.; Zahedi, S.; Ismail, S. et al. 3D printing of PEEK-cHAp scaffold for medical bone implant. *Bio-Des. Manuf.* **2021**, 4, 44-59.

387. Oladapo, B.; Zahedi, S.; Ismail, S. Mechanical performances of hip implant design and fabrication with PEEK composite. *Polymer.* **2021**, 227.

388. Oladapo, B.; Zahedi, S.; Ismail, S.; Olawade, D. Recent advances in biopolymeric composite materials: Future sustainability of bone-implant. *Renew. Sust. Energ. Rev.* **2021**, 150.

389. Oladapo, B.; Zahedi, S.; Omigbodun, F. A systematic review of polymer composite in biomedical engineering. *Eur. Polym. J.* **2021**, 154.

390. Olate, S.; Uribe, F.; Huentequeo-Molina, C.; Goulart, D.R.; Sigua-Rodriguez, E.A.; Alister, J.P. Mandibular Angle Contouring Using Porous Polyethylene Stock or PEEK-based Patient Specific Implants. A Critical Analysis. *J. Craniofac. Surg.* **2021**, 32, 242-246.

391. Omari, A.; Frendø, M.; Sørensen, M.S.; Andersen, S.A.W.; Frithioff, A. The cutting edge of customized surgery: 3D-printed models for patient-specific interventions in otology and auricular management—a systematic review. *Eur. Arch. Oto-Rhino-Laryngol.* **2022**, 279, 3269-3288.

392. Oskolkov, A.; Bezukladnikov, I.; Trushnikov, D. Indirect Temperature Measurement in High Frequency Heating Systems. *Sensors.* **2021**, 21.

393. Othman, H.; Evans, S.G.; Morris, D.; Bhatia, S.; Hayhurst, C. 3D printed models for surgical planning and reconstructive implant design in sphenoorbital tumor surgery. *J. Neurol. Surg. Part B Skull Base*. 2016, 77.

394. Ozlati, A.; Movahedi, M.; Tamizi, M.; Tartifzadeh, Z.; Alipour, S. An alternative additive manufacturing-based joining method to make Metal/Polymer hybrid structures. *J. Manuf. Proc.* **2019**, 45, 217-226.

395. Pagliarulo, V.; Russo, P.; Behal, J. et al. Full-field optical analysis of 3D printing composite for defect detection. proceedings of the SPIE. **2022**, 12137, id. 121370K .

396. Pagliarulo, V.; Russo, P.; Leone, G.; D'Angelo, G.; Ferraro, P. A multimodal optical approach for investigating 3D-printed

carbon PEEK composites. *Opt. Lasers Eng.* **2022**, 151.

397. Palacios-Ibáñez, B.; Relinque, J.J.; Moreno-Sánchez, D. et al. Synthesis and Characterisation of ASA-PEEK Composites for Fused Filament Fabrication. *Polymers (Basel)*. **2022**, 14.

398. Panayotov, I.V.; Orti, V.; Cuisinier, F.; Yachouh, J. Polyetheretherketone (PEEK) for medical applications. *J. Mater. Sci. Mater. Med*. **2016**, 27, 118.

399. Panesar, S.; Belo, J.; D'Souza, R. Feasibility of Clinician-Facilitated Three-Dimensional Printing of Synthetic Cranioplasty Flaps. *World Neurosurgery.* **2018**, 113, E628-E637.

400. Papaefstathiou, S.; Larochette, N. et al. Three-dimensional Printing of Biomimetic Titanium Mimicking Trabecular Bone Induces Human Mesenchymal Stem Cell Proliferation: An In-vitro Analysis. *Spine*. **2022**, 47, 1027-1035.

401. Park, K.; Kim, G.; No, H.; Jeon, H.; Kremer, G. Identification of Optimal Process Parameter Settings Based on Manufacturing Performance for Fused Filament Fabrication of CFR-PEEK. *Appl. Sci*. **2020**, 10.

402. Park, K.; Chahal, P.; Wiwatcharagoses, N. IEEE Computer Soc. *2016 IEEE 66th Electronic Components and Technology Conference (ECTC)*. **2016**, 2239-2244.

403. Park, M.E.; Shin, S.Y. Three-dimensional comparative study on the accuracy and reproducibility of dental casts fabricated by 3D printers. *J. Prosthet. Dent*. **2018**, 119, 861.e1-861.e7.

404. Park, S.; Fu, K. Polymer-based filament feedstock for additive manufacturing. *Comp. Sci. Technol.* **2021**, 213.

405. Park, S.; Lee, J.; Park, J.; Lee, N.; Son, Y.; Park, S. High-temperature 3D printing of polyetheretherketone products: Perspective on industrial manufacturing applications of super engineering plastics. *Mater. Des.* **2021**, 211.

406. Patel, A.; Venoor, V.; Yang, F.; Chen, X.; Sobkowicz, M. Evaluating poly(ether ether ketone) powder recyclability for selective laser sintering applications. *Polym. Degrad. Stab.* **2021**, 185.

407. Peek, N.; Moyer, I. Popfab: A Case for Portable Digital Fabrication. *TEI 2017*. **2017**, 325-329.

408. Perez-Martin, H.; Mackenzie, P.; Baidak, A.; Bradaigh, C.; Ray, D. Crystallinity studies of PEKK and carbon fibre/PEKK composites: A review. *Comp. Part B Eng.* **2021**, 223.

409. Petersmann, S.; Spoerk, M.; Van De Steene, W. et al. Mechanical properties of polymeric implant materials produced by extrusion-based additive manufacturing. *J. Mech. Behav. Biomed. Mater*. **2020**, 104, 103611.

410. Peterson, A. Review of acrylonitrile butadiene styrene in fused filament fabrication: A plastics engineering-focused perspective. *Addit. Manuf.* **2019**, 27, 363-371.

411. Phanindra Bogu, V.; Ravi Kumar, Y.; Kumar Khanra, A. Homogenous scaffold-based cranial/skull implant modelling and structural analysis-unit cell algorithm-meshless approach. *Med. Biol. Eng. Comput*. **2017**, 55, 2053-2065.

412. Picard, M.; Mohanty, A.; Misra, M. Recent advances in additive manufacturing of engineering thermoplastics: challenges and opportunities. *RSC Advances.* **2020**, 10, 36058-36089.

413. Pigliaru, L.; Paleari, L.; Bragaglia, M.; Nanni, F.; Ghidini, T.; Rinaldi, M. Poly-ether-ether-ketone-Neodymium-iron-boron bonded permanent magnets via fused filament fabrication. *Synthetic Metals.* **2021**, 279.

414. Pilloni, G.; Colombo, E.V.; Cofano, F. et al. Custom-made titanium cage to restore the lumbar lordosis: Our experience. *Eur. Spine J*. **2018**, 27, 967-968.

415. Pisula, J.; Budzik, G.; Turek, P.; Cieplak, M. An Analysis of Polymer Gear Wear in a Spur Gear Train Made Using FDM and FFF Methods Based on Tooth Surface Topography Assessment. *Polymers (Basel)*. **2021**, 13.

416. Polyakov, I.; Vaganov, G.; Yudin, V.; Smirnova, N.; Ivankova, E.; Popova, E. Study of Polyetherimide and Its Nanocomposite

3D Printed Samples for Biomedical Application. *Polym. Sci. Series A.* **2020**, 62, 337-342.

417. Pordeus, M.D.; Santiago Junior, J.F.; Venante, H.S.; Bringel da Costa, R.M.; Chappuis Chocano, A.P.; Porto, V.C. Computer-aided technology for fabricating removable partial denture frameworks: A systematic review and meta-analysis. *J. Prosthet. Dent*. **2021**.

418. Pozzi, A.; Arcuri, L.; Lio, F.; Papa, A.; Nardi, A.; Londono, J. Accuracy of complete-arch digital implant impression with or without scanbody splinting: An in vitro study. *J. Dent*. **2022**, 119, 104072.

419. Prechtel, A.; Reymus, M.; Edelhoff, D.; Hickel, R.; Stawarczyk, B. Comparison of various 3D printed and milled PAEK materials: Effect of printing direction and artificial aging on Martens parameters. *Dent. Mater*. **2020**, 36, 197-209.

420. Prechtel, A.; Stawarczyk, B.; Hickel, R.; Edelhoff, D.; Reymus, M. Fracture load of 3D printed PEEK inlays compared with milled ones, direct resin composite fillings, and sound teeth. *Clin. Oral Investig*. **2020**, 24, 3457-3466.

421. Prochor, P.; Mierzejewska, Ż.A. Influence of the Surface Roughness of PEEK GRF30 and Ti6Al4V SLM on the Viability of Primary Human Osteoblasts Determined by the MTT Test. *Materials (Basel)*. **2019**, 12.

422. Przekora, A.; Kazimierczak, P.; Wojcik, M.; Chodorski, E.; Kropiwnicki, J. Mesh Ti6Al4V Material Manufactured by Selective Laser Melting (SLM) as a Promising Intervertebral Fusion Cage. *Int. J. Mol. Sci*. **2022**, 23.

423. Pu, J.; McIlroy, C.; Jones, A.; Ashcroft, I. Understanding mechanical properties in fused filament fabrication of polyether ether ketone. *Addit. Manuf.* **2021**, 37.

424. Pu, Z.; Tu, J.; Han, R. et al. A flexible enzyme-electrode sensor with cylindrical working electrode modified with a 3D nanostructure for implantable continuous glucose monitoring. *Lab. Chip*. **2018**, 18, 3570-3577.

425. Pyon, R.; Zhang, A.; Lin, A. Surgical outcomes of cranioplasty procedures with 3D-printed implants: a systematic review. *Cleft Palate-Craniofac. J.* **2022**, 59, 49-50.

426. Qu, H.; Zhang, W.; Li, Z. et al. Influence of Thermal Processing Conditions on Mechanical and Material Properties of 3D Printed Thin-Structures Using PEEK Material. *Int. J. Precis. Eng. Manuf.* **2022**, 23, 689-699.

427. Rahman, K.; Letcher, T.; Reese, R. Mechanical Properties of Additively Manufactured PEEK Components Using Fused Filament Fabrication. *ASME IMECE 2015, At: Houston, Texas.* **2016**.

428. Ramadan, R.; Elsherbeeny, Y.; Thabet, Y.; Kandil, B.; Ghali, R. Retention of a telescopic overdenture on customized abutments after the simulation of 1 year in function. *Dent. Med. Prob.* **2021**, 58, 201-206.

429. Rekowska, N.; Huling, J.; Brietzke, A. et al. Thermal, Mechanical and Biocompatibility Analyses of Photochemically Polymerized PEGDA(250) for Photopolymerization-Based Manufacturing Processes. *Pharmaceutics.* **2022,** 14.

430. Ren, J.; Paxton, N.C.; Hammond, J. et al. Novel resin tissue array system reduces sample preparation time, labour and reagent costs in bone tissue histology. *Bone*. **2021**, 153.

431. Ren, L.; Wu, W. et al. 3D Printing of Auxetic Metamaterials with High-Temperature and Programmable Mechanical Properties. *Adv. Mater. Technol.* **2022**.

432. Rinaldi, M.; Ferrara, M.; Pigliaru, L.; Allegranza, C.; Nanni, F. Additive manufacturing of polyether ether ketone-based composites for space application: a mini-review. *CEAS Space J*. **2021**.

433. Rinaldi, M.; Ghidini, T.; Cecchini, F.; Brandao, A.; Nanni, F. Additive layer manufacturing of poly (ether ether ketone) via FDM. *Comp. Part B Eng*. **2018**, 145, 162-172.

434. Rinaldi, M.; Ghidini, T.; Nanni, F. Fused filament fabrication of polyetheretherketone/multiwalled carbon nanotube nanocomposites: the effect of thermally conductive nanometric filler on the printability and related properties. *Polym. Int.* **2021**, 70, 1080-1089.

435. Rinaldi, M.; Cecchini, F.; Pigliaru, L.; Ghidini, T.; Lumaca, F.; Nanni, F. Additive Manufacturing of Polyether Ether Ketone (PEEK) for Space Applications: A Nanosat Polymeric Structure. *Polymers (Basel)*. **2020**, 13.

436. Robinson, T.; Hutmacher, D.; Dalton, P. The Next Frontier in Melt Electrospinning: Taming the Jet. *Adv. Funct. Mater.* **2019**, 29.

437. Rodzen, K.; Harkin-Jones, E.; Wegrzyn, M.; Sharma, P.; Zhigunov, A. Improvement of the layer-layer adhesion in FFF 3D printed PEEK/carbon fibre composites. *Comp. Part A Appl. Sci. Manuf.* **2021**, 149.

438. Rodzeń; K.; McIvor, M.J.; Sharma, P.K. et al. The Surface Characterisation of Fused Filament Fabricated (FFF) 3D Printed PEEK/Hydroxyapatite Composites. *Polymers (Basel)*. **2021**, 13.

439. Rodzeń, K.; Sharma, P.K.; McIlhagger, A. et al. The Direct 3D Printing of Functional PEEK/Hydroxyapatite Composites via a Fused Filament Fabrication Approach. *Polymers (Basel)*. **2021**, 13.

440. Rolley, N.; Bonnin, M.; Lefebvre, G. et al. Galenic Lab-on-a-Chip concept for lipid nanocapsules production. *Nanoscale*. **2021**, 13, 11899-11912.

441. Rong, C.; Changlin, D.; Guodong, W. et al. Comparison of 3D-printed Titanium Alloy and Polyether Ether Ketone Prosthetic Beaks for an Injured Red-crowned Crane (Grus japonensis). *J. Avian Med. Surg*. **2022**, 35, 445-450.

442. Roskies, M.; Fang, D.; Abdallah, M. et al. Three-Dimensionally Printed Polyetherketoneketone Scaffolds With Mesenchymal Stem Cells for the Reconstruction of Critical-Sized Mandibular Defects. *Laryngoscope*. **2017**, 127, E392-E398.

443. Roskies, M.; Jordan, J.O.; Fang, D. et al. Improving PEEK bioactivity for craniofacial reconstruction using a 3D printed scaffold embedded with mesenchymal stem cells. *J. Biomater. Appl*. **2016,** 31, 132-139.

444. Rouway, M.; Nachtane, M.; Tarfaoui, M. et al. 3D printing: rapid manufacturing of a new small-scale tidal turbine blade. *Int. J. Adv. Manuf. Technol.* **2021**, 115, 61-76.

445. Rowe, D. Hierarchical surface roughness produced with additive manufacturing technology significantly increases osteogenic cellular differentiation and gene expression when compared to PEEK and smooth titanium surfaces. *Spine J*. **2020**, 20, S150-S151.

446. Russo, P.; Langella, A.; Leone, G. et al. Mechanical and morphological characterization of 3D-printed carbonPEEK composite for avionic shimming. *2021 IEEE 8th International Workshop on Metrology for AeroSpace (MetroAeroSpace).* **2021**, 205-209.

447. Rutkunas, V.; Borusevicius, R.; Liaudanskaite, D. et al. The Effect of Different Cleaning Protocols of Polymer-Based Prosthetic Materials on the Behavior of Human Gingival Fibroblasts. *Int. J. Environ. Res. Public Health*. **2020**, 17.

448. Sagandira, C.; Siyawamwaya, M.; Watts, P. 3D printing and continuous flow chemistry technology to advance pharmaceutical manufacturing in developing countries. *Arabian J. Chem.* **2020**, 13, 7886-7908.

449. Sahal, M.; Chen, M.T.; Sharma, S.; Nair, S.S.; Nair, V.G. 3DP materials and methods for orthopedic, dental and maxillofacial implants: A brief comparative report. *J. 3D Print. Med.* **2019**, 3, 127-134.

450. Santiago, C.; Yelamanchi, B.; de la Pena, J. et al. Thermoplastic Extrusion Additive Manufacturing of High-Performance Carbon Fiber PEEK Lattices. *Crystals.* **2021**, 11.

451. Sarabia-Vallejos, M.; Rodriguez-Umanzor, F.; Gonzalez-Henriquez, C.; Rodriguez-Hernandez, J. Innovation in Additive Manufacturing Using Polymers: A Survey on the Technological and Material Developments. *Polymers*. **2022**, 14.

452. Sarangi, D. A versatile material for dental applications: PEEK. *Indian J. Public Health Res. Dev*. **2019**, 10, 1057-1059.

453. Schmitz, D.; Ecco, L.; Dul, S. et al. Electromagnetic interference shielding effectiveness of ABS carbon-based composites manufactured via fused deposition modelling. *Mater.Today Comm.* **2018**, 15, 70-80.

454. Schönhoff, L.M.; Mayinger, F.; Eichberger, M.; Reznikova, E.; Stawarczyk, B. 3D printing of dental restorations: Mechanical properties of thermoplastic polymer materials. *J. Mech. Behav. Biomed. Mater*. **2021**, 119, 104544.

455. Schönhoff, L.M.; Mayinger, F.; Eichberger, M.; Lösch, A.; Reznikova, E.; Stawarczyk, B. Three-dimensionally printed and milled polyphenylene sulfone materials in dentistry: Tensile bond strength to veneering composite resin and surface properties after different pretreatments. *J. Prosthet. Dent*. **2022**, 128, 93-99.

456. Semeniuk, O.; Cherpak, A.; Robar, J. Design and evaluation of 3D printable patient-specific applicators for gynecologic HDR brachytherapy. *Med. Phys*. **2021**, 48, 4053-4063.

457. Shahriar, B.; France, C.; Valerie, N.; Arthur, C.; Christian, G. Toward improvement of the properties of parts manufactured by FFF (Fused Filament Fabrication) through understanding the influence of temperature and rheological behaviour on the coalescence phenomenon. AIP Conference Proceedings. **2017**, 1896, 040008.

458. Sharma, G.; Vuppuluri, A.; Suresh, K. Essential work of fracture studies of 3D Printed PEEK (Poly-ether-ether-ketone) polymer. *Eng. Fracture Mech.* **2022**, 271.

459. Sharma, N.; Aghlmandi, S.; Cao, S.; Kunz, C.; Honigmann, P.; Thieringer, F.M. Quality Characteristics and Clinical Relevance of In-House 3D-Printed Customized Polyetheretherketone (PEEK) Implants for Craniofacial Reconstruction. *J. Clin. Med*. **2020**, 9.

460. Sharma, N.; Aghlmandi, S.; Dalcanale, F. et al. Quantitative Assessment of Point-of-Care 3D-Printed Patient-Specific Polyetheretherketone (PEEK) Cranial Implants. *Int. J. Mol. Sci*. **2021**, 22.

461. Sharma, N.; Welker, D.; Aghlmandi, S. et al. A Multi-Criteria Assessment Strategy for 3D Printed Porous Polyetheretherketone (PEEK) Patient-Specific Implants for Orbital Wall Reconstruction. *J. Clin. Med*. **2021**, 10.

462. Shely, A.; Livne, S.; Ben-Izhack, G. et al. The Influence of Laboratory Scanner Versus Intra-Oral Scanner on Determining the Implant Axis by Using Three Different Scan Abutments. *Appl. Sci.* **2021**, 11.

463. Shetty, V.; Menon, A.; Sharma, N.; Boootwala, F. Digital Mapping of a Massive Skull-Base Ameloblastoma with Intracranial Extension, Resection, and Reconstruction Using 3D Templates and Molds: Descriptive Case Report and Review. *Asian J. Oncol*. **2022**, 8, 104-110.

464. Shi, H.; Yin, X.; Hu, Y. Solitary Neurofibroma of the Zygoma: Three-Dimensional Virtual Resection and Patient-Specific Polyetheretherketone Implant Reconstruction. *J. Craniofac. Surg*. **2022**.

465. Shi, X.; Chen, B.; Tuo, X.; Gong, Y.; Guo, J. Study on performance characteristics of fused deposition modeling3D-printed composites by blending and lamination. *J. Appl. Polymer Science.* **2021**, 138.

466. Shi, Y.; Liu, J.; Du, M. et al. Customized Barrier Membrane (Titanium Alloy, Poly Ether-Ether Ketone and Unsintered Hydroxyapatite/Poly-l-Lactide) for Guided Bone Regeneration. *Front. Bioeng. Biotechnol.* **2022**, 10, 916967.

467. Shishkovsky, I.; Scherbakov, V.; Kuznetsov, M. Gradient core-shell HTS/polymer covered magnetoactive composites with ultrafine particles fabricated by 3D printing. *Opt.Quantum Electron*. **2016**, 48.

468. Shuchen, D.; Tian, Z.; Ma, C. et al. Establishment of microinvasive modle of chronic rotator cuff injury in rats. *Zhongguo Xiu Fu Chong Jian Wai Ke Za Zhi*. **2014,** 28, 1225-1230.

469. Sikder, P.; Challa, B.; Gummadi, S. A comprehensive analysis on the processing-structure-property relationships of FDM-based 3-D printed polyetheretherketone (PEEK) structures. *Materiallia*. **2022**, 22.

470. Sikder, P.; Ferreira, J.A.; Fakhrabadi, E.A. et al. Bioactive amorphous magnesium phosphate-polyetheretherketone composite filaments for 3D printing. *Dent. Mater*. **2020**, 36, 865-883.

471. Singh, R.; Singh, G.; Singh, J.; Kumar, R. Investigations for tensile, compressive and morphological properties of 3D printed functional prototypes of PLA-PEKK-HAp-CS. *J. Thermoplastic Comp. Mater.* **2021**, 34, 1408-1427.

472. Singh, R.; Singh, J.; Singh, G.; Kumar, R. Friction Stir Spot Welding of PLA-PEKK-HAp-CS Based 3D Printed Scaffolds for Minor Repair. *J. Chin. Soc. Mech. Eng.* **2020**, 41; 639-646.

473. Singh, R.; Singh, G.; Singh, J.; Kumar, R.; Rahman, M.M.; Ramakrishna, S. Thermomechanical investigations of PEKK-HAp-CS composites. *Proc. Inst. Mech. Eng. H.* **2019**, 233, 1196-1203.

474. Singh, S.; Prakash, C, Ramakrishna S. 3D printing of polyether-ether-ketone for biomedical applications. *European Polymer Journal.* **2019**, 114, 234-248.

475. Singh, S.; Prakash, C.; Wang, H.; Yu, X.; Ramakrishna, S. Plasma treatment of polyether-ether-ketone: A means of obtaining desirable biomedical characteristics. *Eur. Polym. J.* **2019**, 118, 561-577.

476. Singh, S.; Singh, G.; Prakash, C.; Ramakrishna, S. Current status and future directions of fused filament fabrication. *J. Manuf. Proc.* **2020**, 55, 288-306.

477. Slosar, P.J. Spine Implant Surface Technology State of the Art. *Spine*. **2018**, 43, S10-S11.

478. Smith, J.A.; Li, S.; Mele, E.; Goulas, A.; Engstrøm, D.; Silberschmidt, V.V. Printability and mechanical performance of biomedical PDMS-PEEK composites developed for material extrusion. *J. Mech. Behav. Biomed. Mater*. **2021**, 115.

479. Smith, J.; Mele, E.; Rimington, R. et al. Polydimethylsiloxane and poly(ether) ether ketone functionally graded composites for biomedical applications. *J.Mech. Behav. Biomed. Mater.* **2019**, 93, 130-142.

480. Smith, K.E.; Dupont, K.M.; Safranski, D.L. et al. Use of 3D printed bone plate in novel technique to surgically correct hallux valgus deformities. *Tech. Orthop*. **2016**, 31, 181-189.

481. Sommacal, S.; Matschinski, A.; Drechsler, K.; Compston, P. Characterisation of void and fiber distribution in 3D printed carbon-fiber/PEEK using X-ray computed tomography. *Comp. Part A Appl. Sci.Manuf.* **2021**, 149.

482. Song, X.; Shi, D.; Song, P.; Han, X.; Wei, Q.; Huang, C. Fused deposition modeling of poly(ether ether ketone) scaffolds. *High Temp. Mater. Process.* **2021**, 40, 1-11.

483. Spagnuolo, G.; Sorrentino, R. The Role of Digital Devices in Dentistry: Clinical Trends and Scientific Evidences. *J. Clin. Med*. **2020**, 9.

484. Spece, H.; DeSantis, P.M.; Kurtz, S.M. Development of an architecture-property model for triply periodic minimal surface structures and validation using material extrusion additive manufacturing with polyetheretherketone (PEEK). *J. Mech. Behav. Biomed. Mater*. **2022**, 133.

485. Spece, H.; Yu, T.; Law, A.W.; Marcolongo, M.; Kurtz, S.M. 3D printed porous PEEK created via fused filament fabrication for osteoconductive orthopaedic surfaces. *J. Mech. Behav. Biomed. Mater*. **2020**, 109, 103850.

486. Stefaniak, A.B.; Du Preez, S.; Du Plessis, J.L. Additive Manufacturing for Occupational Hygiene: A Comprehensive Review of Processes, Emissions, & Exposures. *J. Toxicol. Environ. Health B Crit. Rev*. **2021**, 1-50.

487. Stefanovic, B.; Michalikova, M.; Bednarcikova, L.; Trebunova, M.; Zivcak, J. Innovative approaches to designing and manufacturing a prosthetic thumb. *Prosthet. Orthot. Int.* **2021**, 45, 81-84.

488. Stepashkin, A.; Chukov, D.; Senatov, F.; Salimon, A.; Korsunsky, A.; Kaloshkin, S.; 3D-printed PEEK-carbon fiber (CF) composites: Structure and thermal properties. *Comp. Sci. Technol.* **2018**, 164, 319-326.

489. Strano, M.; Rane, K.; Farid, M.A.; Mussi, V.; Zaragoza, V.; Monno, M. Extrusion-based additive manufacturing of forming and molding tools. *Int. J. Adv. Manuf. Technol*. **2021**, 117, 2059-2071.

490. Sun, F.; Shen, X.; Zhou, N. et al. A speech bulb prosthesis for a soft palate defect with a polyetherketoneketone (PEKK) framework fabricated by multiple digital techniques: A clinical report. *J. Prosthet. Dent.* **2020**, 124, 495-499.

491. Sun, Q.; Shan, Z.; Zhan, L. et al. Warp deformation model of polyetheretherketone composites reinforced with carbon fibers in additive manufacturing. *Mater. Res. Express.* **2021**, 8.

492. Sun, X.; Cao, L.; Ma, H. et al. Experimental Analysis of High Temperature PEEK Materials on 3D Printing Test. *2017 9th*

International Conference on Measuring Technology and Mechatronics Automation (ICMTMA). **2017**, 13-16.

493. Syam, W.; Mannan, M.; Al-Ahmari, A. Rapid prototyping and rapid manufacturing in medicine and dentistry This paper presents an overview of recent developments in the field of rapid prototyping and rapid manufacturing with special emphasis in medicine and dentistry. *Virtual Phys. Prototyp.* **2011**, 6, 79-109.

494. Tan, D.K.; Maniruzzaman, M.; Nokhodchi, A. Advanced pharmaceutical applications of hot-melt extrusion coupled with fused deposition modelling (FDM) 3D printing for personalised drug delivery. *Pharmaceutics*. **2018**, 10.

495. Tan, E.T.W.; Ling, J.M.; Dinesh, S.K. The feasibility of producing patient-specific acrylic cranioplasty implants with a low-cost 3D printer. *J. Neurosurg*. **2016**, 124, 1531-1537.

496. Tang, J.; Kwan, T.; Wu, X. Extrusion and thermal control design of an on-orbit 3D printing platform. *Adv. Space Res.* **2022**, 69, 1645-1661.

497. Tasopoulos, T.; Chatziemmanouil, D.; Kouveliotis, G.; Karaiskou, G.; Wang, J.; Zoidis, P. PEEK Maxillary Obturator Prosthesis Fabrication Using Intraoral Scanning, 3D Printing, and CAD/CAM. *Int. J. Prosthodont.* **2020**, 33, 333-340.

498. Tasopoulos, T.; Kouveliotis, G.; Karoussis, I.; Rfa Silva, N.; Zoidis, P. A Full Digital Workflow for the Duplication of an Existing Implant Retained Overdenture Prosthesis: A Novel Approach. *J. Prosthodont*. **2021**, 30, 555-560.

499. Tavakoli, J.; Diwan, A.D.; Tipper, J.L. Advanced strategies for the regeneration of lumbar disc annulus fibrosus. *Int. J. Mol. Sci*. **2020**, 21, 1-20.

500. Thavasiappan, K.; Venkatesan, M.S.; Ariffuddeen, M.; Ponnuchamy, O.; Ravichandran, N.; Murugesan, G. Design, analysis, fabrication and testing of pc porous scaffolds using rapid prototyping in clinical applications. *Biomedicine*. **2019**, 39, 339-345.

501. Thayaparan, G.K.; Owbridge, M.G.; Thompson, R.G.; D'Urso, P.S. Designing patient-specific solutions using biomodelling and 3D-printing for revision lumbar spine surgery. *Eur. Spine J*. **2019**, 28, 18-24.

502. Thiele, O.C.; Nolte, I.M.; Mischkowski, R.A. et al. Craniomaxillofacial patient-specific CAD/CAM implants based on cone-beam tomography data – A feasibility study. *J. Cranio-Maxillofac. Surg*. **2018**, 46, 1461-1464.

503. Tian, L.; Liu, Y.; Shao, X.; Xu, F.; Wang, X.; Liu, Y. Primary clinical applications of a novel customized polyetheretherketone (PEEK)tmj implant based on 3D printing technology. *Int. J. Oral Maxillofac. Surg*. **2019**, 48, 179-180.

504. Toplosky, V.; Betts, S.; Goddard, R.; Torres, J.; Nguyen, D.; Han, K. Mechanical and Thermal Properties of Glass Reinforced Composites. *IEEE Transactions on Applied Superconductivity.* **2022**, 32.

505. Tsai, P.I.; Wu, M.H.; Li, Y.Y. et al. Additive-manufactured Ti-6Al-4 V/Polyetheretherketone composite porous cage for Interbody fusion: bone growth and biocompatibility evaluation in a porcine model. *BMC Musculoskelet. Disord*. **2021**, 22, 171.

506. Tseng, J.; Liu, C.; Yen, Y. et al. Screw extrusion-based additive manufacturing of PEEK. *Mater. Des.* **2018**, 140, 209-221.

507. Vaezi, M.; Yang, S. Extrusion-based additive manufacturing of PEEK for biomedical applications. *Virtual Phys. Prototyp.* **2015**, 10, 123-135.

508. Vaezi, M.; Zhong, G.; Kalami, H.; Yang, S. Extrusion-based 3D printing technologies for 3D scaffold engineering. In: Deng Y, Kuiper J, eds. *Functional 3D Tissue Engineering Scaffolds: Materials, Technologies and Applications.* **2018**, 235-254.

509. Vaezi, M.; Yang, S. Novel bioactive peek composites produced using 3D printing and dry powder printing technologies. *BioImpacts*. **2018**, 8, 52-53.

510. Vaezi, M.; Black, C.; Gibbs, D.M.R. et al. Characterization of New PEEK/HA Composites with 3D HA Network Fabricated by Extrusion Freeforming. *Molecules*. **2016**, 21.

511. Valentan, B.; Kadivnik, Z.; Brajlih, T.; Anderson, A.; Drstvensek, I. Processing Poly(ether etherketone) on a 3D Printer for Thermoplastic Modelling. *Materiali in Tehnologije.* **2013**, 47, 715-721.

512. van de Vijfeijken, S.E.C.M.; Münker, T.J.A.G.; Spijker, R. et al. Autologous Bone Is Inferior to Alloplastic Cranioplasties: Safety of Autograft and Allograft Materials for Cranioplasties, a Systematic Review. *World Neurosurg*. **2018**, 117, 443-452.e8.

513. Van Horn, M.R.; Beard, R.; Bucklen, B. A roughened 3D-printed surface enhances stem cell proliferation and osteoblast differentiation. *Spine J.* **2020**, 20, S150.

514. Van Horn, M.R.; Beard, R.; Cunningham, B.W.; Mullinix, K.; Allall, M.; Bucklen, B. A biologically-inspired 3D-printed titanium alloy interbody spacer promotes osseointegration in an ovine lumbar interbody fusion model. *Spine J.* **2020**, 20, S34.

515. Van Horn, M.R.; Beard, R.; Wang, W.; Cunningham, B.W.; Mullinix, K.; Bucklen, B. Biomimetic 3D-printed titanium-alloy interbody spacers demonstrate uniform bone growth over 12 weeks. *Spine J.* **2020**, 20, S95-S96.

516. Van Horn, M.R.; Beard, R.; Wang, W. et al. Comparison of 3D-printed titanium-alloy, standard titanium-alloy, and PEEK interbody spacers in an ovine model. *Spine J.* **2021**, 21, 2097-2103.

517. Vasquez, J.; Twigg-Smith, H.; O'Leary, J.; Peek, N. Jubilee: An Extensible Machine for Multi-tool Fabrication. *Proceedings of the 2020 CHI Conference on Human Factors in Computing Systems.* **2020**, 1-13.

518. Vassallo, M.; Rochman, A. Rapid Prototyping Solution for the Production of Vulcanized Rubber Components. *AIP Conference Proceedings.* **2019**, 2113, 150014.

519. Vellone, V.; De Tomaso, S.; Marianetti, T.M.; Sabelli, V.; Ramieri, V. Use of Submental Intubation in the Full-Face Makeover (Orthognathic Surgery, Facial Prosthesis Combined With Rhinoplasty). *J. Craniofac. Surg*. **2022**.

520. Velu, R.; Vaheed, N.; Ramachandran, M.; Raspall, F. Experimental investigation of robotic 3D printing of high-performance thermoplastics (PEEK): a critical perspective to support automated fibre placement process. *Int. J. Adv. Manuf. Technol.* **2020**, 108, 1027-1027.

521. Verma, R.; Virk, S.; Qureshi, S. Interbody Fusions in the Lumbar Spine: A Review. *HSS J.* **2020**, 16, 162-167.

522. Verma, S.; Sharma, N.; Kango, S.; Sharma, S. Developments of PEEK (Polyetheretherketone) as a biomedical material: A focused review. *Eur. Polym. J.* **2021**, 147.

523. Vogel, D.; Weissmann, V.; Ruhrmund, L.; Hansmann, H.; Bader, R. Influence of nozzle temperature and volumetric filling on the mechanical properties of 3D-printed PEEK. *Mater. Test.* **2020**, 62, 351-356.

524. Volyanski, I.; Volchkov, S.; Shishkovsky, I. Cytotoxicity and apoptotic effects of polymer coated copper oxide nanoparticles synthesized via SLM in mesenchymal stem cells. *Opt. Quantum Electron.* **2017**, 49.

525. Wachter, J.; Elsner, C.; Moritzer, E. Investigation of Specific FDM Process Parameters to Optimize the Polymer Discharge of Carbon Fiber Reinforced PEEK. *Macromol. Symp.* **2021**, 395, 2000269.

526. Walsh, W.; Pelletier, M.; Wang, T.; Lovric, V.; Morberg, P.; Mobbs, R. Does implantation site influence bone ingrowth into 3D-printed porous implants? *Spine J.* **2019**, 19, 1885-1898.

527. Wang, H.; Chenn P.; Wu, H. et al. Comparative evaluation of printability and compression properties of poly-ether-ether-ketone triply periodic minimal surface scaffolds fabricated by laser powder bed fusion. *Addit. Manuf.* **2022**, 57.

528. Wang, J.; Gu, J.T.; Meng, M.; Wang, C.Y.; Chen, J.H.; Niu, L.N. Polyetheretherketone Framework for Implant-supported Full-arch Fixed Dental Prostheses in a Periodontitis Patient with a 6-year Follow-up: a Case Report. *Chin. J. Dent. Res*. **2022**, 25, 149-158.

529. Wang, L.; Huang, L.; Li, X. et al. Three-Dimensional Printing PEEK Implant: A Novel Choice for the Reconstruction of Chest Wall Defect. *Ann. Thorac. Surg*. **2019**, 107; 921-928.

530. Wang, L.; Liu, X.; Jiang, T.; Huang, L. Three-dimensional printed polyether-ether-ketone implant for extensive chest wall reconstruction: A case report. *Thorac. Cancer*. **2020**, 11, 2709-2712.

531. Wang, P.; Pan, A.; Xia, L.; Cao, Y.; Zhang, H.; Wu, W. Effect of process parameters of fused deposition modeling on mechanical properties of poly-ether-ether-ketone and carbon fiber/poly-ether-ether-ketone. *High Perf. Polym.* **2022**, 34, 337-351.

532. Wang, P.; Zhang, H.; Cao, Y.; Pan, A.; Wu, W. Additively manufactured short carbon fiber reinforced polyetheretherketone by coating polyetherimide at the interface using fused filament fabrication. *J. Appl. Polym. Sci.* **2022**, 139.

533. Wang, P.; Zou, B. Improvement of Heat Treatment Process on Mechanical Properties of FDM 3D-Printed Short- and Continuous-Fiber-Reinforced PEEK Composites. *Coatings*. **2022**, 12.

534. Wang, P.; Zou, B.; Ding, S. Modeling of surface roughness based on heat transfer considering diffusion among deposition filaments for FDM 3D printing heat-resistant resin. *Appl. Therm. Eng.* **2019**, 161.

535. Wang, P.; Zou, B.; Ding, S. et al. Preparation of short CF/GF reinforced PEEK composite filaments and their comprehensive properties evaluation for FDM-3D printing. *Comp. Part B Eng.* **2020**, 198.

536. Wang, P.; Zou, B.; Ding, S.; Li, L.; Huang, C. Effects of FDM-3D printing parameters on mechanical properties and microstructure of CF/ PEEK and GF/PEEK. *Chin. J Aeronautics.* **2021**, 34, 236-246.

537. Wang, P.; Zou, B.; Ding, S.; Zhuang, Y.; Liu, J.; Li, L. Functionally graded polyetheretherketone-based composites additively manufactured by material extrusion using a transition interface design method. *Comp. Part A Appl. Sci. Manufact.* **2022**, 158.

538. Wang; P.; Zou, B.; Xiao, H.; Ding, S.; Huang, C. Effects of printing parameters of fused deposition modeling on mechanical properties, surface quality, and microstructure of PEEK. *J. Mater. Process. Technol.* **2019**, 271, 62-74.

539. Wang, R.; Cheng, K.; Advincula, R.; Chen, Q. On the thermal processing and mechanical properties of 3D-printed polyether ether ketone. *Mrs Communications.* **2019**, 9, 1046-1052.

540. Wang, Y.; Qu, X.; Jiang, J.; Sun, J.; Zhang, C.; He, Y. Aesthetical and Accuracy Outcomes of Reconstruction of Maxillary Defect by 3D Virtual Surgical Planning. *Front. Oncol*. **2021**, 11.

541. Wang, Y.; Zhang, S.; Nie, B.; Qu, X.; Yue, B. Approaches to Biofunctionalize Polyetheretherketone for Antibacterial: A Review. *Front. Bioeng. Biotechnol*. **2022**, 10, 895288.

542. Wang, Y.; Müller, W.D.; Rumjahn, A.; Schwitalla, A. Parameters Influencing the Outcome of Additive Manufacturing of Tiny Medical Devices Based on PEEK. *Materials (Basel)*. **2020**, 13.

543. Wang, Y.; Müller, W.D.; Rumjahn, A.; Schmidt, F.; Schwitalla, A.D. Mechanical properties of fused filament fabricated PEEK for biomedical applications depending on additive manufacturing parameters. *J. Mech. Behav. Biomed. Mater*. **2021**; 115, 104250.

544. Warburton, A.; Girdler, S.J.; Mikhail, C.M.; Ahn, A.; Cho, SK. Biomaterials in spinal implants: A review. *Neurospine*. **2020**, 17, 101-110.

545. Wei, X.; Zhou, W.; Tang, Z. et al. Magnesium surface-activated 3D printed porous PEEK scaffolds for in vivo osseointegration by promoting angiogenesis and osteogenesis. *Bioact. Mater*. **2023**, 20, 16-28.

546. Weiss, K.; Bagrets, N.; Lange, C.; Goldacker, W.; Wohlgemuth, J. Thermal and mechanical properties of selected 3D printed thermoplastics in the cryogenic temperature regime. *IOP Conf. Ser.: Mater. Sci. Eng.* **2015**, 102, 012022.

547. Weksler, B. Commentary: Three-dimensional printing and customizable implants: The future is now. *JTCVS Tech*. **2021**, 8, 216-217.

548. Westover, B. Three-Dimensional Custom-Root Replicate Tooth Dental Implants. *Oral Maxillofac. Surg. Clin. North America.* **2019**, 31, 489.

549. Weyhrich, C.; Long, T. Additive manufacturing of high-performance engineering polymers: present and future. *Polymer Int.* **2022**, 71, 532-536.

550. Whyte, D.J.; Rajkhowa, R.; Allardyce, B.J.; Wang, X.; Kouzani, A.Z. Design and implementation of an organic powder printer. *Bioprinting*. **2021**, 23.

551. Williamson, W.; Peek, M.; Breads, P.; Coop, B.; Card, G. Tools for Rapid High-Resolution Behavioral Phenotyping of Automatically Isolated Drosophila. *Cell Reports.* **2018**, 25, 1636.

552. Wojnarowska, W.; Miechowicz, S.; Kudasik, T. Effect of manufacturing technique on material homogeneity of an implant made of polyetheretherketone. *Polimery*. **2020**, 65, 771-775.

553. Wong, K.I.; Zhong, Y.; Li, D.; Cheng, Z.; Yu, Z.; Wei, M. Modified porous microstructure for improving bone compatibility of poly-ether-ether-ketone. *J. Mech. Behav. Biomed. Mater*. **2021**, 120, 104541.

554. Wu, B.Y.; Ye, K.; Chen, J.H. et al. Biocompatibility of 3D printed polyetheretherketone/hydroxyapatite composites. *Chin. J. Tissue Eng. Res*. **2023**, 27, 1932-1937.

555. Wu, C.; Zeng, B.; Deng, J. et al. Custom design and biomechanical clinical trials of 3D-printed polyether ether ketone femoral shaft prosthesis. *J. Biomed. Mater. Res. B Appl. Biomater*. **2022**, 110, 2006-2014.

556. Wu, W.; Geng, P.; Li, G.; Zhao, D.; Zhang, H.; Zhao, J. Influence of Layer Thickness and Raster Angle on the Mechanical Properties of 3D-Printed PEEK and a Comparative Mechanical Study between PEEK and ABS. *Materials (Basel)*. **2015**, 8, 5834-5846.

557. Wu, W.; Geng, P.; Zhao, J.; Zhang, Y.; Rosen, D.; Zhang, H. Manufacture and thermal deformation analysis of semicrystalline polymer polyether ether ketone by 3D printing. *Mater. Res. Innov.* **2014**, 18, 12-16.

558. Wu, Y.; Cao, Y.; Wu, Y.; Li, D. Neutron Shielding Performance of 3D-Printed Boron Carbide PEEK Composites. *Materials (Basel)*. **2020**, 13.

559. Wu, Y.; Cao, Y.; Wu, Y.; Li, D. Mechanical Properties and Gamma-Ray Shielding Performance of 3D-Printed Poly-Ether-Ether-Ketone/Tungsten Composites. *Materials (Basel)*. **2020**, 13.

560. Xepapadeas, A.B.; Weise, C.; Frank, K. et al. Technical note on introducing a digital workflow for newborns with craniofacial anomalies based on intraoral scans - part I: 3D printed and milled palatal stimulation plate for trisomy 21. *BMC Oral Health*. **2020**, 20, 20.

561. Xiao, F.; Zhai, Y.; Zhou, Y. et al. Low-temperature fabrication of titania layer on 3D-printed PEKK for enhancing biocompatibility. *Surf. Coat. Technol.* **2021**, 416.

562. Xie, W.; Lorenz, M.; Poosch, F. et al. 3D-printed lightweight dorsal skin fold chambers from PEEK reduce chamber-related animal distress. *Sci. Rep*. **2022**, 12, 11599.

563. Xu, Q.; Chen, Z.; Zhang, Y. et al. Mussel-inspired bioactive 3D-printable poly(styrene-butadiene-styrene) and the in vitro assessment of its potential as cranioplasty implants. *J. Mater. Chem. B*. **2022**, 10, 3747-3758.

564. Xu, Q.; Shang, Y.; Jiang, Z. et al. Effect of molecular weight on mechanical properties and microstructure of 3D printed poly(ether ether ketone). *Polym. Int*. **2021**, 70, 1065-1072.

565. Xu, Q.; Xu, W.; Yang, Y. et al. Enhanced interlayer strength in 3D printed poly (ether ether ketone) parts. *Addit. Manuf.* **2022**, 55.

566. Xu, X.; Wang, H.; Zhang, S. et al. ECM-inspired 3D printed polyetherimide scaffold with Arg-Gly-Asp peptides for the improvement of bioactivity and osteogenic differentiation of osteoblasts. *Mater. Today Comm.* **2022**, 30.

567. Xu, Y.; Xiaofeng, Z.; Detai, Q. et al. Changes in cervical sagittal balance after three-dimensional printing act titanium cage in anterior cervical discectomy with fusion. *Chin. J. Tissue Eng. Res*. **2020**, 24, 5714-5748.

568. Yadav, P.R.; Munni, M.N.; Campbell, L. et al. Translation of polymeric microneedles for treatment of human diseases: Recent trends, Progress, and Challenges. *Pharmaceutics*. **2021**, 13.

569. Yahamed, A.; Ikonomov, P.; Fleming, P.; Pekarovicova, A.; Gustafson, P. Designed structures for bone replacement. *J. Print Media Technol. Res.* **2016**, 5, 291-307.

570. Yang, B.; Chen, W.; Xin, R. et al. Pomelo Peel-Inspired 3D-Printed Porous Structure for Efficient Absorption of Compressive Strain Energy. *J. Bionic Eng.* **2022**, 19, 448-457.

571. Yang, C.; Tian, X.; Li, D.; Cao, Y.; Zhao, F.; Shi, C. Influence of thermal processing conditions in 3D printing on the crystallinity and mechanical properties of PEEK material. *J. Mater. Process. Technol.* **2017**, 248, 1-7.

572. Yang, C.; Wang, B.; Li, D.; Tian, X. Modelling and characterisation for the responsive performance of CF/PLA and CF/PEEK smart materials fabricated by 4D printing. *Virtual Phys. Prototyp.* **2017**, 12, 69-76.

573. Yang, C.; Xu, J.; Xing, Y.; Hao, S.; Ren, Z. Covalent polymer functionalized graphene oxide/poly(ether ether ketone) composites for fused deposition modeling: improved mechanical and tribological performance. *RSC Adv*. **2020**, 10, 25685-25695.

574. Yang, D.; Cao, Y.; Zhang, Z.; Yin, Y.; Li, D. Effects of crystallinity control on mechanical properties of 3D-printed short-carbon-fiber-reinforced polyether ether ketone composites. *Polym. Test.* **2021**, 97.

575. Yang, D.; Yin, Y.; Zhang, Z.; Li, D.; Cao, Y. Wide-angle microwave absorption properties of multilayer metamaterial fabricated by 3D printing. *Mater. Lett.* **2020**, 281.

576. Yang, J.; Xie, J.; Ji, K. et al. Microcellular injection molding of polyether-ether-ketone. *Polymer*. **2022**, 251.

577. Yang, J.; Xie, J.; Ji, K. et al. Effect of mold opening on microcellular polyether-ether-ketone fabricated by injection molding. *J. Mater. Res. Technol.* **2022**, 19, 1678-1689.

578. Yang, W.; Zhao, W.; Li, Q. et al. Fabrication of Smart Components by 3D Printing and Laser-Scribing Technologies. *ACS Appl. Mater. Interfaces*. **2020**, 12, 3928-3935.

579. Yang, X.; Chen, W.; Liu, H. et al. Light-Driven Shape Memory of 3D-Printed PEEK for Programmable Actuations. *Adv. Mater. Technol.* **2022**.

580. Yang, Z.; Liang, Q.; Duan, Y.; Li, Z.; Li, D.; Cao, Y. A 3D-printed lightweight broadband electromagnetic absorbing metastructure with preserved high-temperature mechanical property. *Comp. Struct.* **2021**, 274.

581. Yaragalla, S.; Zahid, M.; Panda, J.K.; Tsagarakis, N.; Cingolani, R.; Athanassiou, A. Comprehensive Enhancement in Thermomechanical Performance of Melt-Extruded PEEK Filaments by Graphene Incorporation. *Polymers (Basel)*. **2021**, 13.

582. Yee-Yanagishita, C.; Essayan, G.; Poojary, B. et al. 16. Evaluation of the cage subsidence performance of three porous lateral cage designs. *Spine J.* **2021**, 21, S8-S9.

583. Yi, N.; Davies, R.; Chaplin, A.; McCutchion, P.; Ghita, O. Slow and fast crystallising poly aryl ether ketones (PAEKs) in 3D printing: Crystallisation kinetics, morphology, and mechanical properties. *Addit. Manuf.* **2021**, 39.

584. Yi, T. A study of conformational variation of temperature-dependent PEEK molecular structures subject to stretching speeds by molecular dynamics simulations. J. *Mech. Sci.Technol.* **2022**, 36, 4143-4151.

585. Yu, D.; Lei, X.; Zhu, H. Modification of polyetheretherketone (PEEK) physical features to improve osteointegration. *J Zhejiang Uni Sci B*. **2022**, 23, 189-203.

586. Yu, Y.; Liu, S. Polyetheretherketone for orthopedic applications: a review. *Curr. Opin. Chem. Eng.* **2021**, 32.

587. Yuan, C.S.; Tang, Y.; Xie, H.Q.; Liang, T.T.; Li, H.T.; Tang, K.L. Application of 3 dimension-printed injection-molded polyether ether ketone lunate prosthesis in the treatment of stage III Kienböck's disease: A case report. *World J. Clin. Cases*. **2022**, 10, 8761-8767.

588. Yuan, Z.; Long, T.; Zhang, J. et al. 3D printed porous sulfonated polyetheretherketone scaffold for cartilage repair: Potential

and limitation. *J. Orthop. Translat*. **2022**, 33, 90-106.

589. Zamborsky, R.; Kilian, M.; Jacko, P.; Bernadic, M.; Hudak, R. Perspectives of 3D printing technology in orthopaedic surgery. *Bratislava Med. J.* **2019**, 120, 498-504.

590. Zanjanijam, A.R.; Major, I.; Lyons, J.G.; Lafont, U.; Devine, D.M. Fused Filament Fabrication of PEEK: A Review of Process-Structure-Property Relationships. *Polymers (Basel)*. **2020**, 12.

591. Zhai, H.; Han, G.; Li, L.; Dong, X.; Jiang, Z.; Lou, F. 3D printed polyetheretherketone material for skull defect repair. *Chin. J. Tissue Eng. Res*. **2023**, 27, 380-384.

592. Zhang, C.; Wang, L.; Kang, J.; Fuentes, O.M.; Li, D. Bionic design and verification of 3D printed PEEK costal cartilage prosthesis. *J. Mech. Behav. Biomed. Mater*. **2020**, 103, 103561.

593. Zhang, H.; Wang, Z.; Wang, Y. et al. Biomaterials for Interbody Fusion in Bone Tissue Engineering. *Front. Bioeng. Biotechnol*. **2022**, 10, 900992.

594. Zhang, J.; Yi, Y.; Wang, C.; Ding, L.; Wang, R.; Wu, G. Effect of Acid-Etching Duration on the Adhesive Performance of Printed Polyetheretherketone to Veneering Resin. *Polymers (Basel)*. **2021**, 13.

595. Zhang, J.; Li, D.; Liu, Y. et al. Application of Patient-Specific PEEK Implant for Aesthetic Considerations in Paranasal Augmentation. *J. Craniofac. Surg*. **2022**.

596. Zhansitov, A.; Slonov, A.; Shetov, R. et al. Synthesis and Properties of Polyetheretherketones for 3D Printing. *Fibre Chem.* **2018**, 49, 414-419.

597. Zhao, F.; Li, D.; Jin, Z. Preliminary Investigation of Poly-Ether-Ether-Ketone Based on Fused Deposition Modeling for Medical Applications. *Materials (Basel)*. **2018**, 11.

598. Zhao, Y.; Wang, Z.; Zhao, J.; Hussain, M.; Wang, M. Additive Manufacturing in Orthopedics: A Review. *ACS Biomater. Sci. Eng.* **2022**, 8, 1367-1380.

599. Zhao, Y.; Zhao, K.; Li, Y.; Chen, F. Mechanical characterization of biocompatible PEEK by FDM. *J. Manufact. Process*. **2020**, 56, 28-42.

600. Zheng, J.; Zhao, H.; Ouyang, Z. et al. Additively-manufactured PEEK/HA porous scaffolds with excellent osteogenesis for bone tissue repairing. *Comp. B Eng.* **2022**, 232.

601. Zheng, J.; Dong, E.; Kang, J. et al. Effects of Raster Angle and Material Components on Mechanical Properties of Polyether-Ether-Ketone/Calcium Silicate Scaffolds. *Polymers (Basel)*. **2021**, 13.

602. Zheng, J.; Kang, J.; Sun, C.; Yang, C.; Wang, L.; Li, D. Effects of printing path and material components on mechanical properties of 3D-printed polyether-ether-ketone/hydroxyapatite composites. *J. Mech. Behav. Biomed. Mater*. **2021**, 118, 104475.

603. Zheng, J.; Zhao, H.; Dong, E. et al. Additively-manufactured PEEK/HA porous scaffolds with highly-controllable mechanical properties and excellent biocompatibility. *Mater. Sci. Eng. C Mater. Biol. Appl*. **2021**, 128, 112333.

604. Zhong, D.; Wang, L.; Li, X.; Huang, L. Research Status of the Skeletalre Construction of Chest Wall. *Zhongguo Fei Ai Za Zhi.* **2018**, 21, 273-276.

605. Zhong, G.; Vaezi, M.; Mei, X.; Liu, P.; Yang, S. Strategy for Controlling the Properties of Bioactive Poly-Ether-Ether-Ketone/Hydroxyapatite Composites for Bone Tissue Engineering Scaffolds. *ACS Omega*. **2019**, 4, 19238-19245.

606. Zhong, G.; Vaezi, M.; Liu, P.; Pan, L.; Yang, S. Characterization approach on the extrusion process of bioceramics for the 3D printing of bone tissue engineering scaffolds. *Ceram. Int*. **2017**, 43, 13860-13868.

607. Zhong, L.; Jin, H.; Chen, D. et al. Computer-aided design and manufacturing with 3D printing for cranio-orbital defect

reconstruction and artificial eye implantation: single case report and literature review. *Int. J. Clin. Exp. Med.* **2019**, 12, 9446-9452.

608. Zhong, R.; Xie, Z.; Liao, Y.; Li, Y.; Huang, C. Clinical application of triangular parabolic PEEK mesh with hole button produced by combining CAD, FEM and 3DP into cranioplasty. *Biomed. Res*. **2018**, 29, 2703-2710.

609. Zhou, M.; Si, L.; Chen, P. et al. Experimental investigation and numerical simulations of temperature and morphology in material extrusion additive manufacturing. *Int. J. Adv. Manufact. Technol.* **2022**, 119, 4863-4876.

610. Zhou, T.; Zhou, Y.; Hua, Z. et al. 4D printing high temperature shape-memory poly(ether-ether-ketone). *Smart Mater. Struct.* **2021**, 30.

611. Zhou, Z.; Yan, G.; Wang, Z. et al. Inhibition of hyperplasia during the implantation of the puborectalis-like artificial anal sphincter. *Int. J. Artif. Organs*. **2020**, 43, 482-493.

612. Zhu, C.; He, M.; Sun, D. et al. 3D-Printed Multifunctional Polyetheretherketone Bone Scaffold for Multimodal Treatment of Osteosarcoma and Osteomyelitis. *ACS Appl. Mater. Interfaces*. **2021**, 13, 47327-47340.

613. Zhukauskas, R.; Horvath, S.; Zhukauskas, A. Osseointegration with pekk 3D technology tetrafuse in an ovine bony defect model. *Tissue Eng. Part A*. **2017**, 23, S146.

614. Zivcak, J.; Hudak, R.; Schnitzer, M.; Kula, T. Numerical Simulation and Experimental Testing of Topologically Optimized PLA Cervical Implants Made by Additive Manufacturing Methodics. *Acta Mech.* **2018**, 12, 141-144.
